# Advances in Screening, Early Diagnosis and Accurate Staging of Diabetic Neuropathy

**DOI:** 10.3389/fendo.2021.671257

**Published:** 2021-05-26

**Authors:** Josie Carmichael, Hassan Fadavi, Fukashi Ishibashi, Angela C. Shore, Mitra Tavakoli

**Affiliations:** ^1^ Diabetes and Vascular Research Centre, National Institute for Health Research, Exeter Clinical Research Facility, University of Exeter Medical School, Exeter, United Kingdom; ^2^ Peripheral Neuropathy Group, Imperial College, London, United Kingdom; ^3^ Internal Medicine, Ishibashi Medical and Diabetes Centre, Hiroshima, Japan

**Keywords:** microvascular complications, Diabetic Neuropathy, Screening, Diagnosis, Early Detection, neuropathy biomarkers

## Abstract

The incidence of both type 1 and type 2 diabetes is increasing worldwide. Diabetic peripheral neuropathy (DPN) is among the most distressing and costly of all the chronic complications of diabetes and is a cause of significant disability and poor quality of life. This incurs a significant burden on health care costs and society, especially as these young people enter their peak working and earning capacity at the time when diabetes-related complications most often first occur. DPN is often asymptomatic during the early stages; however, once symptoms and overt deficits have developed, it cannot be reversed. Therefore, early diagnosis and timely intervention are essential to prevent the development and progression of diabetic neuropathy. The diagnosis of DPN, the determination of the global prevalence, and incidence rates of DPN remain challenging. The opinions vary about the effectiveness of the expansion of screenings to enable early diagnosis and treatment initiation before disease onset and progression. Although research has evolved over the years, DPN still represents an enormous burden for clinicians and health systems worldwide due to its difficult diagnosis, high costs related to treatment, and the multidisciplinary approach required for effective management. Therefore, there is an unmet need for reliable surrogate biomarkers to monitor the onset and progression of early neuropathic changes in DPN and facilitate drug discovery. In this review paper, the aim was to assess the currently available tests for DPN’s sensitivity and performance.

## Introduction

Diabetes is one of the fastest-growing health challenges of the 21^st^ century, with the number of adults living with diabetes having more than tripled over the past 20 years (1). The International Diabetes Federation reported that in 2019, the prevalence of diabetes was 9.3% (463 million people worldwide) with a predicted rise to 10.9% (700 million people) by 2045 (2). Furthermore, it has been shown that over 1.1 million children and adolescents below 20 years have type 1 diabetes. On top of these staggering figures, are the number of people with impaired glucose tolerance (IGT) or metabolic syndrome with 373.9 million in 2019 (7.5%) and predicted rise to 548.4 million (8.6%) by 2045 (2).

In the UK alone, there were 4.8 million people with diabetes in 2019. Diabetes is on the rise. Figures from Diabetes UK shows that someone is diagnosed with diabetes every two minutes, with 5.3 million expected to be living with the condition by 2025 (3).

Diabetes is strongly associated with both microvascular and macrovascular complications. As a result, 10% of global health expenditure, equal to USD 760 billion, is directed toward diabetes and its complications (2). Microvascular changes lead to nephropathy, retinopathy and neuropathy. Among these complications, diabetic peripheral neuropathy (DPN) is the most common and costly diabetes-associated complication, occurring in around 50% of individuals with diabetes (4). Distal symmetric polyneuropathy (DSPN) (5) typically follows a distal-proximal course and results in symmetrical symptoms and signs between the body’s left and right sides. Common symptoms include burning, numbness, tingling, pain and/or weakness starting in the distal lower extremities which progress into more extreme symptoms of neuropathic pain in around 10-30% of affected patients (6, 7). Symptoms may be sporadic or constant but can be debilitating and in many people lead to depression, sleep disorders and overall reduced quality of life (8).

The true prevalence of DPN is underestimated as its assessment is challenging. However, DPN is recognized as the most common complication of diabetes.

DPN is the strongest initiating risk factor for diabetic foot ulceration (neuropathic ulcer) (9, 10), and existing ulcers may be further exacerbated from damage to sensory neurones. Resultant limb numbness causes ulcers to remain undetected for longer periods (10); thus, corrective actions are not taken nor advice sought at early stages of the disease. Often the first sign that a person has diabetic peripheral neuropathy (DPN) is a foot ulcer, which may lead to irreversible tissue damage, lower limb amputation and significant morbidity.

In the UK, people with diabetes account for more than 40% of hospitalizations for major amputations and 73% of emergency admissions for minor amputations. A single diabetes related foot ulcer can take over 240 days to put into remission and costs £8,000 pa to treat. Ulcers frequently recur and eventually may require the amputation of a lower limb. DPN is hugely costly to our NHS (>£1.1 billion pa in direct medical costs) and to the wider UK economy (~£4 billion), is particularly debilitating and distressing for patients and their families and can lead to an untimely death (11, 12), with five-year mortality ranging from 52 to 80 percent after major amputation (13).

Furthermore, with diabetes related lower limb amputations increasing at the rate of almost 20% per annum in line with the increasing prevalence of diabetes, there is a huge strain on NHS budgets which are unable to keep up.

Autonomic neuropathies are a class of DPN which share similar diffuse pathophysiology with DSPN, but differ by being largely non-sensory (4). These typically affect the cardiovascular, urogenital and gastrointestinal systems. Patients may also suffer from sudomotor dysfunction, hypoglycemia obliviousness, and abnormal pupillary function (5). Rare forms of DPN include mononeuropathies, polyradiculopathies and treatment-induced neuropathies (5). These atypical forms are generally self-limiting and resolve with medical management and physical therapy, usually over several months (11).

In clinical settings, there are several different approaches to assess diabetic peripheral neuropathy (DPN), and the choice of the test will depend on the aim of testing. It is usually sufficient in a busy clinic to establish whether a patient is symptomatic, particularly of painful DPN (12), and whether or not they are at high risk of foot ulceration typically through monofilament testing. However, to fully assess damage and phenotype of DPN, sensory deficits must be detected early. Those accurate biomarkers are available for monitoring of DPN and for use in clinical trials of potential new treatments.

Currently, there are no simple markers for early detection of DPN in routine clinical practice. The measures we use are crude and detect the disease very late in its natural history. Even the benefits gained by standardizing clinical assessment with scored clinical evaluations remain subjective, heavily reliant on the examiners’ interpretations.

This paper reviews the current knowledge and the optimal approaches for diagnosis and screening of diabetic peripheral neuropathy.

## Types of Nerve Fibers

Peripheral nerve fibers can be classified using Erlanger and Gasser’s classification, which defines nerves based on diameter, conduction speed, and myelination level (**Table 1**). A-fibers have the largest diameter, with the thickest myelination and fastest conduction speed, and act as sensory and motor fibers within the somatic nervous system. They may be further divided into large nerve fibers that have sensory and motor functions (Aα and Aβ), and small nerve fibers (Aγ which has motor functions, and Aδ which may be autonomic or sensory fibers) (14).

**Table 1:** Classification of nerve fibers in the peripheral nervous system according to modified Erlanger and Gasser.

Classification	Myelination	Diameter (um)	Conduction Velocity (m/s)	Type	Function
**Aα (alpha)**	Yes	12-22	70-120	Sensory/motor	Proprioception, touch sensory, somatic motor to extrafusal muscles
**Aβ (beta)**	Yes	5-12	30-70	Sensory/motor	Proprioception, touch/pressure sensory, somatic motor to intrafusal muscles
**Aδ (delta)**	Yes	1-5	5-30	Sensory	Touch and cold thermoreceptors, nociception
**Aγ (gamma)**	Yes	2-8	15-30	Motor	Somatic motor to intrafusal muscles
**B**	Yes	<3	3-15	Autonomic	Visceral afferent fibers and preganglionic efferent fibers
**C**	No	0.1-1.3	0.6-2	Sensory/ autonomic	Temperature (warm receptors), pain perception, nociception, itching

Group B-fibers are small, with moderate myelination and slower conduction velocities than A-fibers. B-fibers act mainly as general visceral afferent and pre-ganglionic fibers and are found only in the autonomic nervous system.

Group C-fibers have a small diameter, low conduction velocity and are the only unmyelinated group. They act as somatic, afferent fibers that carry sensory information relating to temperature and pain, as well as having autonomic functions such as the stimulation of the sweat glands (14).

### Epidemiology

The prevalence of diabetic peripheral neuropathy (DPN) reported in various studies ranges from 6% to 51% depending on the population (15, 16). In the Diabetes Control and Complications Trial/Epidemiology of Diabetes Interventions and Complications (DDCT/EDIC) Study, the prevalence of DPN in adults with type 1 diabetes was 6% at baseline and increased to 30% after 13–14 years of follow-up (17). The prevalence of DPN among adults with type 1 diabetes in the Pittsburgh Epidemiology of Diabetes Complications was 34% and increased significantly with age (18–29 years: 18%; ≥30 years: 58%). It has been estimated that half of all children with diabetes with a duration of 5 years or longer already have diabetic neuropathy (18) and nearly 25% of pediatric patients with newly diagnosed diabetes have abnormal findings on nerve conduction studies (NCS), indicating nerve damage (19).

The prevalence of DPN is somewhat higher in patients with T2DM when compared to T1DM (4). The ‘Action to Control Cardiovascular Risk in Diabetes’ (ACCORD) (20) trial and the ‘Veteran Affairs Diabetes Trial’ (21) found that DPN was present in 42% and 39% of adults with type 2 diabetes, respectively, at baseline measurement. A study comparing magnetic resonance imaging (MRI) scans of the sciatic nerve in T1DM, and T2DM patients with DPN found that the predominant type of nerve lesion differed between the two (22). This study found that in T1DM, lesions were predominantly associated with poor glycemic control and loss of nerve conduction, whereas in T2DM lesions were associated with changes in lipid metabolism. This raises the question of whether damage to peripheral nerves results in different patterns of nerve damage, and thus would require different types of preventive treatment.

#### Risk Factors

In both main types of diabetes, the prevalence and severity of DPN increases with disease duration and increasing age (16). A large study of 1172 patients with diabetes assessed for neuropathy at baseline reported that patients who had developed neuropathy by roughly ten-year follow-up were on average 3.8 years older and had diabetes for 3.3 years longer at baseline (16). Furthermore, the study found that in both T1DM and T2DM, higher hemoglobin A1c (HbA1c) level was a significant predictor of the development of diabetic neuropathy (16).

In cohorts of patients with T2DM, several metabolic syndromes such as hypertension, abdominal obesity, lower high-density lipoprotein (HDL) levels and hypertriglyceridemia have been consistently associated with DPN development (23), with additional independent risk factors including alcohol abuse and increased height (24). In a cohort of patients with T1DM, the EURODIAB prospective complications (25) study reported similar modifiable risk factors to those identified in T2DM, explicitly having an association with raised triglyceride level, obesity, smoking and hypertension. Several genes have also been linked to an increased risk of diabetic neuropathy. Still, only ACE (encoding angiotensin-converting enzyme) and MTHFR (encoding methylenetetrahydrofolate reductase) polymorphisms have been confirmed using large patient cohorts in multiple populations (24). Research into the role of genetics in diabetic neuropathy is currently limited, and many more studies are required.

Significantly lower levels of clinical neuropathy in South Asian patients have been reported compared to Europeans and Afro-Caribbean (26). A recent study found that in a population of people with type 2 diabetes, South Asians had significantly better-preserved small nerve fiber integrity than equivalent Europeans (27). However, this patient cohort was recruited from primary care, and most patients had no or mild neuropathy, so it was not representative of the diabetic population overall. A proposed explanation for the reduced risk was the differences in the transcutaneous partial pressure of oxygen (TCpO2) and height between the ethnicities (27). However, the study suggesting this explanation did not adjust for a range of possible confounders such as obesity, and alcohol intake, between ethnicities, all of which are established risk factors for developing DPN. A more recent study suggested that the variation may be due to differences in height and adiposity between the ethnic groups, as the adjustment for these factors rendered the difference insignificant (28).

#### Prevention/Treatment

There is currently no Food and Drug Administration (FDA) approved therapy to prevent or reverse human DPN (4). The current management approach focuses on reasonable glycemic control, lifestyle modifications, and management of associated pain. The reasonable glycemic control consists of not only strict HbA1c control but also reduced glycemic variability, because glycemic variability has recently emerged as an another measure of glycemic control, which might constitute an additive, or even better predictor of microvascular complications including neuropathy than mean HbA1c levels (29, 30).

Previous studies have found that improving HbA1c levels does affect DPN progression in patients with T2DM (20, 31). The ACCORD study (20) found that intensive treatment caused delay in onset of albuminuria and it reduced neuropathy, MNSI socre, loss of ankle jerks, loss of light touch at end of the study. The veterens study (31) assessed whether new evidence of clinical neuropathy occurred during the period of intensive versus normal control and had quite severe criteria for definaitions. The Epidemiology of Diabetes Interventions and Complications (EDIC) trial reported that intensive glucose control significantly delayed the development and progression of diabetic neuropathy in T1DM patients over time (17). Another study, following a cohort of T1DM patients over 24 years confirmed these findings. Patients who had stable, near-normal HbA1c levels (mean <7.0%) had significantly less deterioration in nerve fiber function when measured using electrophysiology and quantitative sensory methods (p<0.05 for all measures at 24 years follow-up) (32).

Attempts have been made to reduce DPN by lifestyle interventions (24). Several studies have demonstrated a potential for improved outcomes in patients with diagnosed DPN through exercise regimes put in place over ten weeks (33) to 12 months (34). Despite insignificant improvements in body mass index (BMI), these studies reported a significant improvement in objective nerve function measures and reduced neuropathy symptoms. Neither of these studies included a control group, which is essential to provide a measure of the change in neuropathy which could be expected over time without intervention but with the same amount of scrutiny, for example, additional contact time with healthcare professionals or individuals paying more attention to their health due to taking part in a study. In the absence of a control group, it is difficult to ensure that neuropathy improvements are genuinely due to modifications in exercise regimes alone.

A recent comprehensive study in Japanese patients with T2DM under poor glycemic control (HbA1c 9.6%, 81.6 mmol/mol) at baseline assessed the impact of intensive glucose control without hypoglycemia and found that normalizing A1c over the two years of follow-up resulted in significant improvement in neuropathy outcomes (35). This study also revealed that small glycemic variability assessed by SD and coefficient of variation (CV) of monthly measured HbA1c levels was associated with the improvement of neuropathy outcomes. In the follow-up study of T2DM (a median of 9.3 years), mean HbA1c levels were the main risk predictor for the composite outcome of developing or worsening diabetic neuropathy, whereas glycemic variability assessed by HbA1c variability was a better risk predictor for new incident of neuropathy (36). In the follow-up study of T1DM the long-term glycemic variability assessed by CV and SD of HbA1c levels was linked to DPN independent of mean HbA1c (37). These studies indicated that the strict HbA1c control as a long-term mean glycemic levels and suppressed glycemic variability are required for the prevention and slow neuropathy progression in patients with diabetes.

#### Screening

The American Diabetes Association (ADA) and the International Working Group on the Diabetic Foot (IWGDF) recommends regular examination of people with DM for the diagnosis of DPN and loss of protective sensation using simple standard tests for the identification of those at risk for diabetic foot ulcer (38, 39). It is recommended that all patients with T2DM be screened for DPN at diagnosis, and for T1DM, the screening should begin five years post-diagnosis (40). After this initial screening, all patients should be reviewed annually.

Nerve conduction studies (NCS) are considered the gold standard for the diagnosis of large fibers neuropathy. The Toronto consensus (41) recommended the use of abnormal NCS with a symptom or sign to diagnose DPN. However, the need for specialist examiners and equipment renders NCS inappropriate as a screening test. Thus, it is used only to confirm any possible/probable DN picked up post-screening using other measures (40).

More commonly, screening for DPN involves history taking for neuropathic symptoms and examination of the feet, along with a screening test (40). Traditional screening tests benefit from being quick and easy; however, like NCS, these only assess larger fiber function and are unable to detect any early changes in small nerve fibers. Furthermore, two systematic reviews focusing on the use of monofilament testing, a commonly used screening test for DPN, described a variation of diagnostic value in the current literature and a lack of consistency in recommended test procedure and interpretation (42). Sensitivity for peripheral nerve fiber damage ranged from 43-93% when using NCS as a reference standard (43). Both review papers did not recommend the sole use of monofilament testing to diagnose peripheral neuropathy (43). This is just one example of the shortcomings of current screening tests.

## Diagnostic Tests for DPN

While there is no single accurate definition of diabetic neuropathy, a simple definition for clinical practice is the presence of symptoms and/or signs of peripheral nerve dysfunction in people with diabetes after excluding other causes (5). Based on this, the diagnostic tests are focused on assessing the symptoms and signs of nerve dysfunctions.

**Figure 1:**
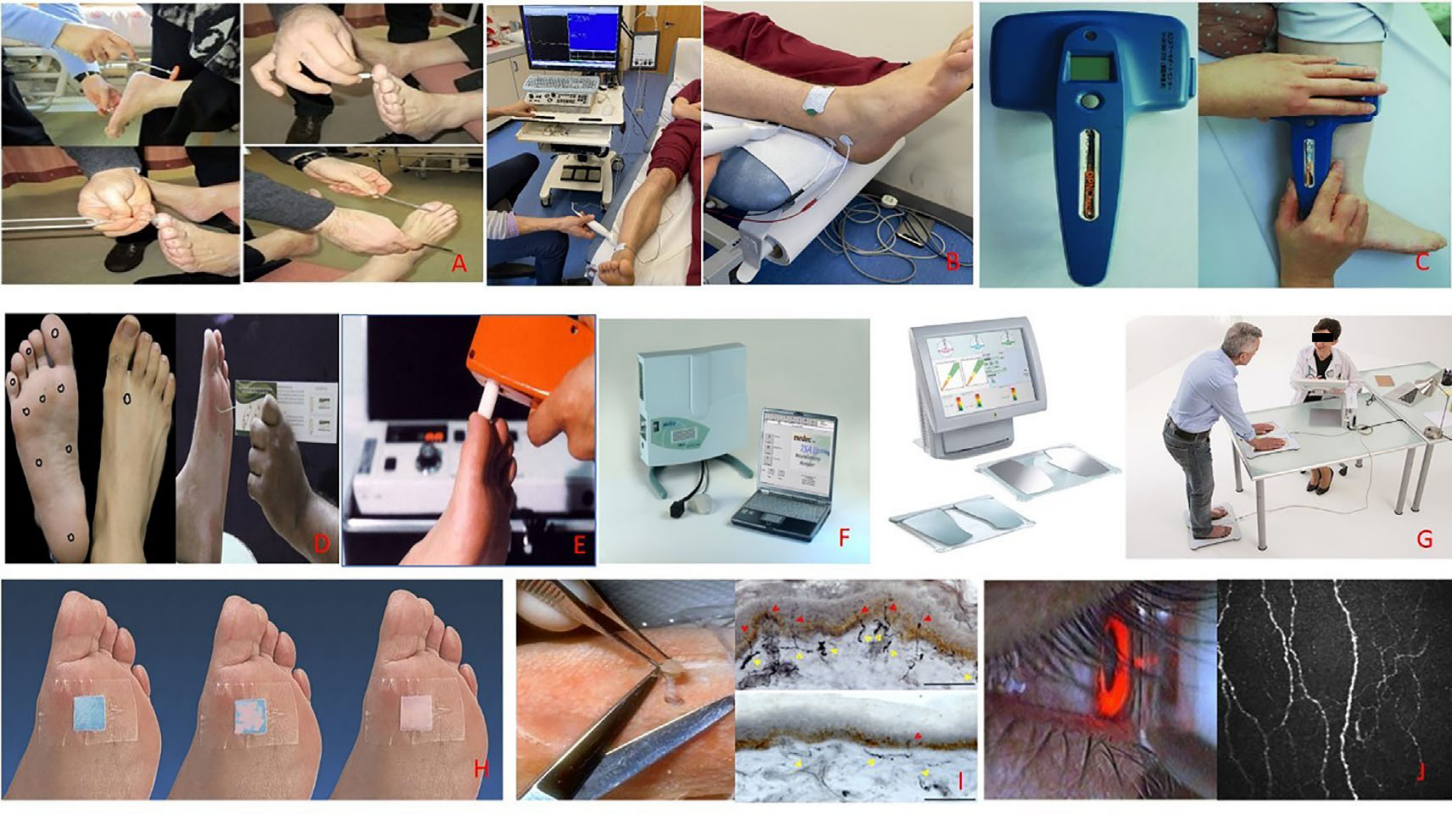
Examples of nine different tests for diabetic peripheral neuropathy (DPN), **(A)** Physical examination and Neuropathy disability score (NDS), **(B)** Nerve Conduction studies being performed on the lower leg, **(C)** DPNcheck device to test sural nerve conduction performed on the lower leg, **(D)** Monofilament screening test sights and procedure, **(E)** Vibration perception threshold testing, **(F)** Medoc TSAII quantitative sensory testing device for thermal perception threshold, **(G)** Sudoscan equipment. Hand and feet sensor plates with displaying test results, **(H)** Neuropad test demonstrated original blue color, **(I)** A punch skin biopsy to collect samples needed for IENFD measurement, **(J)** An image of the corneal sub-basal nerves using corneal confocal microscopy and the CCM probe positioning during corneal scanning.

There are numerous testing methods available to assess the peripheral nervous system’s structure and function, with each test having its own advantages and disadvantages. Bedside tests used to aid diagnosis of DPN—including the 10g monofilament (**Figure 1D**), the Ipswich Touch Test, and vibration perception threshold testing with the Vibratip (**Figure 1E**), a tuning fork, or automated devices such as Neurobiothesiometer, are not only reliant on patients’ subjective response but are also mainly used to identify the loss of protective foot sensation and risk of ulceration. As such, these tests tend to diagnose DPN when it is already well established. Late diagnosis hampers the potential benefits of intensified multifactorial intervention at an early stage of the disease, which could prevent the sequelae of DPN. Unfortunately, by the time DPN is detected with the crude tests currently used, it is often very well established and consequently impossible to reverse or halt the inexorable neuropathic process. Early diagnosis and timely intervention are thus essential in preventing the development of DPN.

Some of the most common tests and methods for diagnosis of DN have been summarized in **Table 2**.

**Table 2:** Diagnostic tests available for assessing DPN.

		Nerve Fibers Assessed	Advantages	Limitations
**Symptoms and Signs**	**Questionnaires**	Large (Aβ-fibers) and Small (Aδ and C-fibers)	Easy to administer. Used for monitoring symptoms (44)	Lack of Sensitivity, accuracy and reproducibility, Subjective (45)
	**NDS**	Large (Aβ-fibers) and Small (Aδ and C-fibers)	Does not require specialist equipment, Assesses large and small-fiber function (46)	Not sensitive or reproducible, Low correlation with small fiber quantitative tests (47)
	**10-gram Monofilament**	Large (Aβ-fibers)	Simple, quick and inexpensive (48).	No standardization of methods. Cannot detect early neuropathy (48).
	**Ipswich Touch Test**	Large (Aβ-fibers)	Simple. Requires no specialist equipment (49).Can test at home	Can only detect advanced neuropathy (48)
	**QST** (Thermal and Vibration thresholds)	Large (Aβ-fibers) and Small (Aδ and C-fibers)	Measures small and large fiber function (44)Good repeatability (50)	Unable to differentiate between peripheral and central abnormalities (51) High inter-operator variability (52)
**Large FiberTests**	**DPNCheck**	Large, sural nerve (Aβ-fibers)	Quick, Easy to perform,Good sensitivity (92-95%) compared to NCS (53, 54)	Relies on the accessibility of sural nerve (55). Validation studies had small patient numbers (53, 54)
	**NCS**	Large (Aβ-fibers)	A sensitive measure of large nerve function (56),Reproducible (57)	Doesn’t assess small fibers, Uncomfortable (58), Does not assess early neuropathic changes
**Small Fiber Tests**	**Skin Biopsy (IENFD)**	Small (C-fibers)	Gold standard for SNF, Quantitative,Good sensitivity,Detects early nerve changes (59, 60)	Invasive, Risk of infection, Repeatability, Requires trained personnel and special labs (44)
	**CCM**	Small (Aδ and C-fibers)	Non-invasive,Good reproducibility (61), Rapid and objective (62, 63)	Relatively Expensive,Requires specialist equipment and personnel,manual analysis is time-consuming (44)
**AutonomicTests**	**Neuropad**	Small (C-fibers)	Can be self-administered,suitable for screeningNon-invasiveGood sensitivity (54, 64–69)	Varied interpretation of the results (54, 64–67)
	**Sudoscan**	Small (C-fibers)	Non-invasive,Easy to perform	Unclear if measuring sudomotor function Variable specificity (53-92%) (70–74)
	**QSART**	Small (C-fibers)	Sensitive for SFN (82%) (75) Gold standard for measuring sudomotor function	Time-consuming, Requires specialist equipment and trained personnel (76) Uncomfortable

IENFD, Intra-epidermal nerve fiber density; NCS, Nerve conduction studies; QSART, Quantitative sudomotor axon reflex test; CCM, Corneal confocal microscopy; NDS, Neuropathy disability score; QST, Quantitative sensory testing; SFN, Small fiber neuropathy.

### Symptoms

Various clinical scoring systems are available for DPN screening which involve symptom scoring, sign scoring or both (**Table 3**). These systems may enhance diagnostic accuracy through a composite score of different combined tests and are useful tools for aiding diagnosis of DPN, along with quantitative measures. Each questionnaire has a scoring system which can diagnose, and in some, stratify disease severity. **Table 3** presents a summary of the most commonly used questionnaires for assessing DPN.

**Table 3:** Summary of questionnaires available for assessing DPN.

	Questionnaire	Assessed	Type of Administration	Scoring
**Symptoms**	NSP	Symptoms of neuropathy	Clinician administered	**34** categories (women) **36** categories (men)
	DNS	Symptoms of diabetic peripheral neuropathy	Clinician administered	4 for Symptoms **(Total 4)**
	NSS	Symptoms of neuropathy	Clinician administered	8 for muscle weakness 5 for sensory disturbances 4 for autonomic symptoms **(Total 17)**
	NPQ	Symptoms of neuropathic pain	Completed by the patient	**Total of 12**
	NPSI	Symptoms of neuropathic pain	Completed by patient	10 descriptors, 2 duration **(Total 12)**
	McGill Pain Questionnaire	Multidimensional symptoms of pain	Clinician administered	Subclass 1- Sensory Subclass 2 - Affective Subclass 3 - Evaluative Subclass 4 - Miscellaneous **(Total 78)**
	CNE	Signs of peripheral neuropathy	Clinician administered	21 for sensory testing 8 for muscle strength 4 for ankle reflex **(Total 33)**
**Signs**	NDS	Signs of peripheral neuropathy	Clinician administered	2 for vibration sensation 2 for temperature sensation 2 for pinprick 4 for ankle reflex **(Total 10)**
	DNE	Signs of peripheral neuropathy	Clinician administered	4 for muscle strength 2 for reflex responses 10 for sensory testing **(Total 16)**
	NIS-LL	Signs of neuropathy in the lower limbs	Clinician administered	64 for muscle strength 16 for sensory testing 8 for reflex responses **(Total 88)**
	MNDS	Signs of peripheral neuropathy	Clinician administered	12 for sensory tests 18 for muscle strength 16 for reflex testing **(Total 46)**
	UENS	Signs of peripheral neuropathy	Clinician administered	11 for each side **(Total 22)**
**Symptoms and** **Signs**	DN4	Symptoms and signs of neuropathic pain	Clinician administered	7 for symptoms 3 for signs **(Total 10)**
	LANSS	Symptoms and signs of neuropathic pain	Clinician administered	5 for symptoms 2 for signs **(Total 7)**
	TCNS	Signs and symptoms of peripheral neuropathy	Clinician administered	6 for symptoms 5 for sensory tests 8 for reflex tests **(Total 19)**
	MNSI	Signs and symptoms of peripheral neuropathy	Symptoms by patient Foot examination by a clinician	15 for symptoms 8 for foot examination **(Total 23)**

NSP, Neuropathy Symptoms Profile; NPQ, Neuropathic Pain Questionnaire; DNS, Diabetic Neuropathy Symptom; NSS, Neuropathy Symptom Score; NPSI, Neuropathic Pain Symptom Inventory; CNE, Clinical Neurological Examination; NDS, Neuropathy Disability Score; DNE, Diabetic Neuropathy Examination; NIS-LL, Neuropathy Impairment Score in the Lower Limbs; MNDS, Michigan Neuropathy Disability Score; UENS, Utah Early Neuropathy Scale; DN4, Douleur Neuropathique en 4; LANSS, Leeds Assessment of Neuropathic Symptoms and Signs; TCNS, Toronto Clinical Neuropathy Score; MNSI, Michigan Neuropathy Screening Instrument (47, 77–86).

The Neurological Symptom Score (NSS) is a 17 question, interview-based assessment of sensory, motor, and autonomic function used to screen DPN (87). Still, it is considered too extensive to be used efficiently in clinical practice. The diabetic neuropathy score (DNS) is an adaptation of the NSS that is a much quicker screening method, with only four questions and still offering moderate sensitivity (79%) and specificity (78%), but with slightly lower reliability for diagnosing DPN (45) when using a diagnostic score of 1 or more.

Other symptom scoring systems focus only on pain and differentiating neuropathic from other causes. Clinicians commonly recognize pain descriptors that are used by patients with neuropathic pain. The McGill pain questionnaire was the first questionnaire designed to offer a multidimensional assessment of pain, which included assessing severity or intensity, emotional impact, and significance to the pain sufferer (88). This questionnaire is one of the most commonly used multi-dimensional pain scales globally. A short-form is available for use in screening which has shown good agreement with the original version (89).

#### Signs

The Neuropathy disability score (NDS) is a commonly used clinical examination method that assesses neuropathy signs (**Figure 1A**). Thirty-five items are used for both sides, evaluating cranial nerve damage, muscle strength, sensation loss and reflex delay/loss (90). However, some of the items have demonstrated a weak relation to DPN, and the full scoring system is too long to be used in clinical practice. Therefore a revised NDS has been created. This system is more commonly used and tests for four main neuropathy signs; ankle reflex, vibration, pinprick and temperature sensation at both sides of the largest toes. A maximum score is 10, and usually, more than 6 is considered abnormal (91).

#### Composite Scoring Systems

The reliance on symptoms or signs alone may lead to low diagnostic accuracy for the presence of DPN, and a combination of both allows a more thorough assessment. Several scoring systems assess both signs and symptoms of DPN to produce a composite score. The Toronto clinical neuropathy score (TCNS) consists of three parts: symptom scores, reflex test scores and sensory test scores. The maximum score is 19, and the test is able to stratify patients into no DPN, mild DPN, moderate DPN and severe DPN depending on the overall score (92). Testing has proven validity and reliability for diagnosing and staging DPN compared to electrophysiology measures (92).

The Michigan neuropathy screening instrument (MNSI) is another commonly used composite scoring system that includes a questionnaire and a foot examination (77). Neuropathy can be defined as seven or more positive responses to this symptoms section alone (77). The foot examination is more frequently used and encompasses foot appearance (including ulcers), ankle reflex and the 128‐Hz tuning fork test (77). One study (93) found a range of sensitivity (35-79%) and specificity (65-94%) in comparison to NCS depending on the cut-off value used for abnormality in MNSI. The higher specificity values indicate a potential high diagnostic impact for MNSI scoring; however, the lower sensitivity range indicates that milder DPN cases are likely not to get picked up.

Scoring of symptoms and signs is convenient and easy to perform as a method of screening for DPN. These tests are easily interpreted, making them a useful tool in supporting decisions on which patients should be referred on for specialist assessment. Quantitative, objective measures should be considered when the patient has signs and symptoms other than those rated by the scoring test.

### Large Fiber Tests

#### Nerve Conduction Studies (NCS)

The current ‘gold standard’ for clinical diagnosis of DPN is through nerve conduction studies (NCS) by a trained neurophysiologist (**Figure 1B**). In 2010, the Toronto Consensus, by an expert panel recommended that one abnormal finding as part of NCS, combined with a symptom or sign of neuropathy should be used to confirm DPN (41). NCS has also demonstrated an ability to predict future DPN (94).

For reliable NCS results, close attention must be paid to factors such as filter setting, limb temperature, and recording location, as outcomes can be vulnerable to variations. Trials have demonstrated that NCS consistently demonstrate excellent intra-observer agreement (58, 95); however, a poor inter-observer agreement between expert clinical neurophysiologists is common (58) when no standardized, specific technique is followed. One study (95) assessed the results of 4 neurophysiologists, from 4 different centers. Specific assessment methods were provided in a specially prepared syllabus, and a training session was provided beforehand. The outcome was a significant improvement in inter-observer agreement with a standardized approach, and although not entirely eliminated, levels of disagreement were consequently considered clinically significant for medical practice (95).

Conversely, when considering the use of NCS in therapeutic clinical trials, even small inter-observer variability may be significant enough to impact results through impacting the statistical power of a study and thus the trial’s outcomes. This may partially explain why previous clinical trials have used NCS as a primary outcome to detect treatment efficacy and have reported failed outcomes (96–98). Evidence supports the use of a single observer to repeat electrophysiological tests on each patient in these trials.

Furthermore, Standard NCS testing is not easily applicable as a screening tool for DPN since it is time-consuming, requires a specialist operator and can be uncomfortable for the patient (58). Electrodiagnostic studies have also been identified as one of the largest drivers of health care costs related to neuropathy evaluation (99). Results are often found to be normal in patients with diabetes who have early or small fiber predominant neuropathy.

#### DPNCheck

To overcome some of the shortcomings of standard NCS testing, a novel point-of-care nerve conduction device (POCD), DPN-Check (Neurometrix Inc., Waltham, MA) has been developed with the potential to serve as an acceptable proxy to standard NCS which are time-consuming, expensive, and often require patients to be seen in specialist clinics (**Figure 1C**). This test for sural nerve conduction velocity and amplitude is much quicker (3 minutes) to perform than conventional electrodiagnostic testing. It has been validated in type 1 and 2 diabetes populations through comparison with the Neuropathy Disability Score (NDS) (55) and standard NCS (53, 54). These studies have reported a high sensitivity of 92-95% for detecting abnormalities (**Table 4**). However, these studies’ cohorts have been small, with two of the three studies assessing very low numbers of patients with type 1 diabetes (53, 54, 108). Furthermore, the DPNCheck device is dependent on the presence of an accessible sural nerve which can be anatomically absent in up to 9% of healthy subjects (55).

**Table 4:** Summary studies for validity for four potential screening tests for DPN.

Test	Fibers Assessed	Validated Against	Sensitivity	Specificity
**DPNCheck** (53, 54)	Large (Aβ-fibers)	NCS	92-95%	82-89%
**Neuropad** (64, 66–69)	Small (C-fibers)	NCS, NDS, VPT	70-97.8%	50-67%
**Sudoscan** (70–74)	Small (C-fibers)	NCS, Clinical Examination, UENS, VPT, NSS	70-87.5%	53-92%
**CCM** (62, 100–107)	Small (Aδ and C-fibers)	NCS, Clinical Examination,CASS	59-86%(CNFL) 65-82%(CNFD) 17-100%(CNBD)	61-84%(CNFL) 41-79%(CNFD) 45-96%(CNBD)

### Small Fiber Tests

#### Punch Skin Biopsy

The evidence strongly suggests that in DPN, damage to small fibers precedes damage to large fibers (109, 110) and punch skin biopsy is currently considered the gold-standard single test for diagnosing small fiber neuropathy (111). A measure of intra-epidermal nerve fiber density (IENFD) can be quantified from these biopsies, which is a method of documenting the density of terminal branches of peripheral nerves within the epidermis (no/mm^2^). The European Federation of Neurological Societies has published guidelines for its use in diagnosing peripheral neuropathies (112) (**Figure 1I**).

Two immuno-staining methods have become the most widely used in IENFD measurement: indirect immunofluorescence (IF) and bright-field immunohistochemistry (BFI). Although IF is considered a slightly more sensitive technique due to higher signal/noise ratio (113), the two methods have excellent correlation (114), and both can comparably detect SFN (113). At present, age-related normative values exist only for BFI, published by a multi-national group of 8 centers (115).

For both IF and BFI techniques, IENFs are typically counted directly through an epifluorescence microscope’s oculars by focusing through the optical planes (113). For IF only, the more precise, but time-consuming technique confocal microscopy (CM) can analyze optical sections of 3-dimensional images using computer software (113). The two techniques have shown excellent correlation (113), and the latter is usually used when the more complex, second-level analysis is needed.

IENFD measurements have been shown to detect small fiber neuropathy with depletion of IENFD detected in patients with normal NCS and no clinical signs or symptoms of neuropathy (59, 60). A recent study reported low sensitivity of just 61% when using a cut off of 4.5fibers/mm IENFD to diagnose clinical DPN in T1DM patients (100). Earlier studies have published significantly higher values for sensitivity (80%) (116) and specificity (95%) (117), however, these studies were comparing healthy controls to DPN patients rather than the test’s ability to identify DPN in a diabetic cohort. Other studies have found a decrease in IENFD correlating with the progression of neuropathy and duration of diabetes (118, 119) with reports that IENFD may also be lower in patients with painful DPN compared to painless DPN (120).

A 5-year follow-up study investigating the progression of DPN in T1DM and T2DM, reported a significant reduction of IENFD in T2DM patients, with IENFD measurement being the single most abnormal parameter (121). Overall, the reduction in IENFD was not significant in T1DM subjects. However, the lower number of patients in the T1DM group may explain this finding, as this would make it more challenging to prove statistically significant changes (121).

The main issue with IENFD measurements as a biomarker for small fiber neuropathy is that it is an invasive procedure. Obtaining a biopsy can cause side effects such as a mild infection due to improper wound management or, less commonly, excessive bleeding. Even though reported side effects are rare (1.9/1000) (115), the nature of this technique limits its practical use, particularly when a repeat biopsy is required in longitudinal studies or clinical intervention trials.

From a screening perspective, although intra-epidermal nerve fiber density measurement from a lower-limb skin biopsy is considered the gold standard for the diagnosis of small fiber neuropathy, it is invasive and therefore not suitable for routine screening (111, 122).

#### Quantitative Sudomotor Axon Reflex Test QSART

The assessment of sudomotor nerve (sweat) function has also been used to assess small autonomic c-fibers, as anhidrosis can be characteristic of the presence of peripheral autonomic neuropathy.

The reference standard for measuring sudomotor function is the quantitative sudomotor axon reflex test (QSART). This test uses local sweat production, measured as a change of relative humidity over time, during and after skin activation. Special software is used to digitalise, plot and analyze the temporal resolution, latency, magnitude and duration of the sudomotor response (123). However, due to highly technical demands and relative discomfort of the examination, QSART remains mostly limited to research centres and is not considered a potential screening tool for DPN (76).

#### Neuropad

Neuropad^®^ is a patented 10-minute screening test for the early detection of diabetic foot syndrome and can be used as a triage test (124). It is a unique, non-invasive, painless and simple diagnostic screening test employing a chemical reaction to minute quantities of sweat as a biomarker for much earlier signs of DPN.

The test has been created to assess the sweat function (small autonomic c-fibers) in the feet of patients with suspected neuropathy. An adhesive pad containing cobalt salts is stuck onto the foot’s plantar aspect and changes color from blue to pink within 10 minutes if the sudomotor function is normal (67). If there is a decreased function, the pad remains blue or turns patchy in color. There is a strong association between skin dryness, sudomotor dysfunction and diabetic foot ulcer and the function of Neuropad. An abnormal Neuropad response is associated with sympathetic dysfunction and clinical neuropathy (**Figure 1H**).

This test’s main advantage is that patients can self-administer at home, reducing clinical contact time and aiming to reinforce abnormal results visually. Instructions have been confirmed as clear for patients to follow, and the test is easy to use for most patients (64). However, due to older age, visual and kinetic problems, a fifth of patients still needed help when self-testing.

It has been reported as having good to excellent (70-97.8%) (64–69) sensitivity for DPN detection (**Table 4**). When comparing Neuropad to a range of different small and large fiber diagnostic tests, strong correlation between Neuropad and NDS (64, 69), IENFD (65), CCM (125), Sudoscan (126) and measures of sweat gland dysfunction (127) have been reported. It has also been identified as a useful tool for staging the severity of neuropathy in patients with type 2 diabetes demonstrating excellent agreement with the Michigan classification system (128). Another significant advantage of Neuropad is its high NPV, making it ideal to serve as a screening test primarily to exclude DPN (68, 129, 130).

However, studies are not consistent in terms of the position of the Neuropad on the foot and the NDS cut-off value chosen to indicate clinical DPN presence. Furthermore, some studies graded the Neuropad color change as a percentage (66) or score out of 1 (65), whereas others simply classified the results as normal or abnormal (64, 67). Standardization of elapsed time before test result analysis is also necessary as extending the observation period to 15 minutes may provide greater diagnostic usefulness (131). This highlights a need for software development that can consistently grade each test’s color change over time to enable continuous and more accurate monitoring of sudomotor dysfunction.

In order to address these issues and increase both the sensitivity and particularly the specificity of Neuropad screening and create a continuous output, a smartphone software app and internet based image processing system has been developed. Neurometrics-Diab™ is a digital therapeutics (DTx) smartphone app which uses the Neuropad™ as a biomarker to produce a continuous record of a person’s neuropathy to see if it is improving, is stable or is worsening with trend-lines helping to predict outcomes. Using a smartphone camera, patients can take a photo of their test result which is then automatically sent to a web server where the photo is run through a proprietary image processing algorithm resulting in a percentage score which is recorded. Over time a trend can be calculated. The DTx app is currently at the advanced prototype stage. Versions for other medical conditions are under development.

#### Sudoscan

Sudoscan™ (Impeto Medical) is another quick, simple and non-invasive test that aims to assess sudomotor function using ‘reverse iontophoresis’ (132, 133) to measure electrochemical skin conductance (ESC) of sweat in the hands and feet. Compared to age-corrected standard data, a reduced ESC result may indicate degeneration of small c-fibers that innervate the sweat glands and, therefore, lead to reduced sweat gland function (71) (**Figure 1G**).

The ESC measurements from the feet are considered more sensitive for the detection of DPN than the hands (72), with less variation in results (134). This is likely due to a fluctuation in the hands’ contact on the electrodes. In contrast, the feet are aided by gravity to maintain constant pressure on the electrodes throughout the test. Lower electrochemical skin conductance at the feet was also significantly associated with increasing symptoms in a large cohort of patients with T2DM (135).

Reference values in healthy subjects are available from a global collaborative analysis comparing different ethnic groups, age, and gender (136). This study noted a significantly lower hands and feet ESC for African-American, Indian, and Chinese populations than the Caucasian population, highlighting the need to match ethnicity groups in electrochemical skin conductance studies. The same study also observed no significant difference between women and men at the hands or feet and a weak decline in ESC with increased age.

ESC measurements may also be associated with subjects’ weight (137), perhaps due to a weight-dependent change in sensitivity of the stainless-steel electrodes, or sweat gland density, when the subject is in the standing position. This could also be due to the correlation between higher weight and larger feet only (137). These hypotheses are yet to be assessed; however, these studies’ findings emphasize the importance of profile matching different subject groups for a weight that did not occur in some validation studies (71, 74).

Validation studies have reported consistently good values for sensitivity (70-87.5%) (**Table 4**) when using foot ESC results to screen for DPN (70–72, 74, 132). However, there are inconsistencies in the ESC cut-off values used for identifying sudomotor dysfunction, ranging from 52uS (70) to 77uS (72). This variation and inconsistencies in the neuropathy tests being used as a reference standard are the likely cause of the extensive range in reported specificity of between 53-92% (70–72, 74). It highlights the need for standardization of the classification criteria used. Patient cohorts also differed in their severity of DPN, with participants in one study (74) having significantly more advanced DPN than those in the study by Smith and colleagues (71). Therefore the test performed better in the former.

Overall, Sudoscan appears to be a promising DPN screening test that is non-invasive, easy to perform and eliminates the subjective component of clinician error, demonstrating good correlation with IENFD (137). However, there is some doubt as the current evidence does not strongly support ESC to distinguish between patients with DPN and control individuals (138). Therefore, longitudinal and more extensive cohort validation studies are needed, along with standardization of diagnostic criteria before Sudoscan can be used as a screening tool for small fiber neuropathy.

It is evident that progress has been made in developing point of care devices (POCDs) which may be capable of diagnosing DPN early before clinical signs are apparent. Neuropad, DPNCheck and Sudoscan are newer screening tests that have demonstrated potential for early detection, however validation studies, thus far, have reported a range of sensitivities, specificities depending on cohort and test used for comparison (**Table 3**).

### Quantitative Sensory Testing (QST)

Quantitative sensory testing (QST) has become a common method for evaluating small nerve fiber function using thermal threshold and thermal pain measurements and large fiber function using vibration thresholds (52). The most common commercial system is the Medoc TSA-II NeuroSensory Analyzer (Medoc Advanced Medical Systems, Israel) which is used to determine thermal thresholds, (**Figure 1F**). In recent years a cheaper, more portable device has been designed, NerveCheck (Phi Med Europe S.L., Barcelona, Spain), which has shown good reproducibility (ICC values = 0.79, 0.71 and 0.86 for vibration, warm and cold sensation respectively) and comparable diagnostic accuracy (86%, 72% and 79% for vibration, warm and cold sensation testing respectively) to established QST equipment for the diagnosis of DPN (139).

Cold thresholds can be used to evaluate myelinated A-delta fiber function, whereas warm thresholds are used to assess the function of unmyelinated C-fibers. Published normative data sets are available for heat threshold detection (140–145), and recommendations for conducting QST in both clinical practice and research have previously been published by The International Association for the Study of Pain (NeuPSIG) (146).

QST has been found to have reasonable repeatability (50); however, inter-operator and inter-patient variability depend on several factors. Training of both examiner and patient, the methodology of assessment, baseline skin temperature, stimulus characteristics, location and number of stimuli sites and duration of intervals between tests have all affected QST measurements (52). Using standardized methodology with extensive training has significantly reduced interobserver variability (147, 148). However, this may be too time-consuming to be implemented.

When it comes to the effects of body fat on thermal detection thresholds, there are conflicting findings. Malmström et al. (149) failed to detect differences between obese and other groups for cold and warm thresholds at the suprailiac site (149). In contrast, Pryce and colleagues (150) found that obese participants had significantly higher cold and warm detection thresholds than normal BMI participants on the abdomen.

Two psychophysical algorithms can be used to determine thermal thresholds. These are the method of limits and the method of level (described in detail elsewhere (50, 151), with the method of limits used more commonly due to it being less time-consuming (146). Measurements determined using limits have been reported as significantly higher than those measured by Level, irrespective of test location (52). However, the two methods correlate well with each other (52) and the 2013 consensus concluded that both were reliable (146). The major difference between these two methods is the effect of reaction time. For the method of limits, a patient has a longer reaction time due to age or height (causing a more extended sensory pathway) which may erroneously give a higher threshold.

Both warm and cold thresholds can be affected in patients with DPN, irrespective of how long the course of diabetes is, but the frequency of abnormal warm thresholds is significantly higher (141). A study found that cold detection thresholds significantly reduced in DM patients with no evidence of pre-clinical, sub-clinical and clinical DPN, respectively (152). A longitudinal study also found a significant positive correlation between deterioration of cold detection thresholds and pain intensity in painful DN, with warm detection thresholds also correlating at non-significant value (153).

One major issue with the use of QST is that it cannot differentiate between peripheral and central temperature perception causes. It involves sensory receptors, spinal cord pathways and termination sites in the thalamus. This means that if there is poor concentration, a language barrier or cognitive defect, subjects’ results may affect their subjective nature (51).

## Corneal Nerves as a Biomarker for DPN

Anatomically and developmentally, the eye can be considered an extension of the central nervous system (CNS). The cornea is the most densely innervated tissue in the body. It is richly supplied by a large number of sensory nerve fibers and a lesser number of autonomic fibers (154). The cornea possesses small unmyelinated C-fibers and myelinated Aδ-fibers for sensory innervation. These are derived from the trigeminal nerve’s ophthalmic division and enter the corneal stroma at its periphery, in a radial fashion parallel to the corneal surface. As the fibers run forward toward the cornea center, they lose their myelin sheath; a necessary step to maintain corneal transparency (154).

Corneal C-fibers form a delicate three-dimensional network known as the ‘sub-basal nerve plexus’ (155), which is located beneath the basal layer of the corneal epithelium. Mapping of the cornea (156) has shown that this plexus forms a spiral or ‘whorl like’ pattern. The spiral center, often called the vortex, is located approximately 2-3 mm inferior and nasal to humans’ corneal apex. Due to this arrangement, sub-basal nerves in the superior and human apical cornea are oriented vertically. In contrast, sub-basal nerves in other corneal regions may be orientated horizontally or obliquely, consistent with their locations within the whorl-like plexus (157)

Corneal confocal microscopy (CCM) is a non-invasive, *in vivo* ophthalmic imaging technique that allows a detailed examination of the cornea, at high magnification, on a cellular level (**Figure 1J**) (158). By capturing multiple two-dimensional images at different depths, CCM imaging can delineate the corneal layers of the cornea (158), providing superior magnification compared to standard slit-lamp biomicroscopy. These properties allow CCM to acquire high-quality images of the corneal C-fibers in the sub-basal nerve plexus. Considering the known relationship between damage to these fibers and diabetic peripheral neuropathy, the potential for their use as a surrogate biomarker for DPN has been identified.

When analyzing the sub-basal nerve plexus, most studies report results from four morphological parameters: Corneal nerve fiber density (CNFD) which is the total number of main nerve fibers per mm^2^, corneal nerve fiber length (CNFL) which is the sum of the length of all nerve fibers and branches (mm/mm^2^), tortuosity coefficient (TC) which is a unitless measurement that uses deviation from a straight line to measure the tortuosity of the main nerve fibers independent of their orientation, and corneal nerve branch density (CNBD) which is defined as the number of branches emanating from all main nerve fibers. There is, however, a discrepancy in how this can be quantified between studies with the established protocol for these parameters described elsewhere (62).

Of these four parameters, CNFL has been the most frequently used parameter for DPN, with one study reporting superior reliability than other parameters (159). Some studies have assessed the diagnostic performance of CCM for DPN and reported the results for CNFL only (101, 105). Hertz et al. (159) reported that CNFL produced the highest intra-observer and inter-observer reproducibility (ICC of 0.72 and 0.73 respectively), with TC demonstrating the lowest (0.23 and 0.29 respectively).

Two other parameters that have been reported in research studies are nerve reflectivity (160) and nerve fiber beading (number/100 µm) (161, 162). Nerve fiber reflectivity is usually assessed using grades as first outlined by Oliveira-Soto and Efron (160), whereby classification can be split into four grades according to a comparison with reference images. The number of beadings is defined as the number of beadings in a length of 100um of sub-basal nerves within a frame (163). Both parameters have demonstrated changes in dry eye conditions, where patients with Sjogren’s syndrome have demonstrated significantly higher beading than dry eye patients of other primary causes (164). However, both measures require subjective judgment. Beading can be challenging to quantify and may require special software and may have poor repeatability and reproducibility (163).

More recently, newer corneal parameters have been investigated. These include inferior whorl length (IWL) (165) defined as the length of the nerves at the inferior whorl of the superficial nerve plexus, nerve fiber width (166) and nerve fiber area (167). These new measures have previously shown significant differences between the non-neuropathic and clinically neuropathic groups in DM (168) with CNFW and CNFA, demonstrating 74% and 66% sensitivity-specificity equal error rate point, respectively when identifying non-neuropathic patients compared to control subjects (168). This indicates that these new measures may have the capacity to identify individuals with early neuropathy; however, research into these new parameters is currently limited.

Another type of cell found in the sub-basal layer and has been of interest in DPN research are dendritic cells. These antigen-presenting cells of the cornea are of paramount importance. They play a critical role in activating other immune systems in the ocular surface, influencing both suppression and induction of inflammation (169, 170).

Langerhans cells are usually up to 15μm in diameter and can be seen in various forms (171). In their immature form, these cells have small dendritic processes or lack dendrites completely and are mainly located in the peripheral cornea’s epithelium (172). In pathological states, Langerhans cells mature, form interlocking dendritic processes which may comprise a net-like structure, and migrate from the periphery into the central cornea (172).

Cross-sectional studies have shown an increase in the densities of Langerhans cells in the central cornea related to conditions such as dry eye with and without contact lens wear (171, 173) bacterial keratitis (174), thyroid eye disease (175) and diabetes (176, 177).

### CCM for the Detection of DPN

In the early 2000s, a novel study by Rosenberg and colleagues reported the correlation between increasing severity of DPN, corneal sensitivity and progressive loss of corneal sub-basal nerve fibers (178). This was closely followed by a similar small study published in 2003 (179) which found that CCM was able to detect abnormalities in the corneal nerves of 18 patients with diabetes deemed to have only mild neuropathy using conventional tests. Similarly, Midena and colleagues (180) reported a significant decrease in corneal nerve fiber and branch number, along with decreased beading in patients with diabetes. It should be noted that these three studies used a light corneal confocal microscope, which is the first commercially available generation of the confocal imaging device with inferior image quality in comparison to the methods now commonly used.

Since then, the use of corneal confocal microscopy (CCM) for rapid, noninvasive clinical assessment of corneal nerves has grown substantially, especially in recent years. It has proven to be particularly useful as a diagnostic marker for detecting diabetic neuropathy and a range of other peripheral neuropathies (62, 100–106, 181–186). Some of them are reviewed in this paper.

#### Diagnostic Performance for Clinical DPN

Several cross-sectional studies have evaluated the ability of CCM to diagnose clinical levels of DPN in comparison to a range of other diagnostic tests (**Table 5**). It must be noted that most of these studies assessed patients with T1DM only, meaning there is limited published data available for the diagnostic sensitivity and specificity values when assessing patients with T2DM.

**Table 5:** Summary of studies assessing the clinical utility of corneal nerve parameters for the diagnosis of clinical levels of diabetic neuropathy compared to chosen reference standards.

	Study	Number subjects	Type of CCM device	Diabetes Type	Age(years)	Disease Duration(years)	Type of Neuropathy	Validated Against	Sensitivity(%)	Specificity(%)	CNFL Threshold(mm/mm²)	CNFD Threshold(no./mm²)	CNBD Threshold (no./mm²)	CNFT(TC)
**Cross-sectional**	Tavakoli et al. (**62**)	118 (101 DM, 17 HC)	Tomey ConfoScan P4	1,2	55 ± 4.8 (HC) 55 ± 1.9(DPN-) 58 ± 2.1(Mild DPN) 59 ± 2.5(Mod DPN) 61 ± 2.05(Sev DPN)	10.7 ± 1.82(DPN-) 15.5 ± 2.08(Mild DPN) 18.6 ± 3.06(Mod DPN) 19.3 ± 2.85(Sev DPN)	DSPN	NDS	64 (CNFL) 82 (CNFD) 91(CNBD)	79 (CNFL) 52 (CNFD) 45 (CNBD)	3.39	27.81	13.89	–
	**Ahmed et al.** (101)	153 (89 DM, 63 HC)	HRT (II)	1	38.9 ± 17.6 (HC) 34.9 ± 14.8 (DPN-) 50.0 ± 14.3 (DPN+)	17.6 ± 14.0(DPN-) 31.4 ± 13.5(DPN+)	DSPN	NCS, Clinical Examination	85	84	14	–	–	–
	Tavakoli et al. (**107**)	52 (34 DM, 18 HC)	HRT (III)	1,2	42 ± 0 (DAN-) 44 ± 3(DAN+)	12 ± 3 (DAN-) 26 ± 2 (DAN+)	DAN	Composite autonomic scoring scale (CASS)	86 (CNFL) 86 (CNFD) 100 (CNBD)	78(CNFL) 79(CNFD) 56 (CNBD)	4.8	23.3	19.5	–
	**Chen et al.** (102)	89 (63 DM, 26 HC)	HRT (III)	1	44 ± 15 (HC) 44 ± 13 (DPN-) 59 ± 11 (DPN+)	23 ± 16 (DPN-) 39 ± 14 (DPN+)	DSPN	NCS, DNS/NDS	59 (CNFL) 82 (CNFD) 17 (CNBD)	74 (CNFL) 71 (CNFD) 96 (CNBD)	16.5	24	15	–
	**Alam et al.** (100)	88 (61 DM, 27 HC)	HRT (III)	1	41 ± 114.9(HC) 38.8 ± 12.5 (DPN-) 53.3 ± 11.9 (DPN+)	17.2 ± 12.0 (DPN-) 37.2 ± 13.1(DPN+)	DSPN	NCS, Clinical Examination	74 (CNFL) 77(CNFD) 67 (CNBD)	61(CNFL) 79 (CNFD) 58 (CNBD)	16.8	25	36.5	–
	**Scarr et al.** (106)	137 (67DM, 69HC)	HRT(III)	1	64 ± 8 (HCs) 65 ± 7 (T1DM)	52-58 (Median 54)	DSPN	NCS, Clinical Examination	73 (CNFL) 76(CNFD) 44 (CNBD)	75 (CNFL) 75(CNFD) 75 (CNBD)	13.7	18.8	15.6	–
	**Perkins et al.** (104)	998	Tomey Confoscan	1	42 ± 19	21 ± 15	DSPN	NCS, Clinical Examination	71 (CNFL) 65 (CNFD) 67 (CNBD)	67 (CNFL) 75 (CNFD) 72 (CNBD)	16.4	28	37.6	–
	(Consortium)	(516 T1DM, 482 T2DM)	P4, HRT (II),HRT (III)	2	62 ± 10	12 ± 9			65 (CNFL) 69 (CNFD) 69(CNBD)	69 (CNFL) 41(CNFD) 63(CNBD)	16.3	39.2	44.8	–
**Longitudinal**	**Pritchard et al.** (105)	90 (T1 DM)	HRT (III)	1	42 ± 16(DPN-) 51 ± 14(DPN+)	15 ± 12(DPN-) 29 ± 16(DPN+)	DSPN	NCS, DNSS/NDS	63	74	14.1	–	–	–
	**Lovblom et al.** (103)	65 (T1 DM)	HRT (III)	1	34 ± 15 (DPN-) 38 ± 16 (DPN+)	17 ± 12 (DPN-) 21 ± 9 (DPN+)	DSPN	NCS, TCNS	82(CNFL) 55(CNFD) 82(CNBD) 73(CNFT)	69 (CNFL) 59(CNFD) 50 (CNBD) 72(CNFT)	14.9	41.7	36.1	15.9

Studies presented are all published studies. Data presented as standard units or mean ± standard deviation.

These studies used a cut-off point for the reference neuropathy test/combination of tests to determine whether a patient had a DPN. However, the reference test and cut-off points varied between studies meaning there were no universal diagnostic reference criteria. Some studies validated CCM against a single test of nerve conduction studies (NCS) (100, 101) or neuropathy disability score (NDS) (62). In contrast, other studies used a combination of the two (102) or NCS and clinical examination (104, 106). A combination of diagnostic tests will likely increase the efficiency of detecting DPN compared to one test used alone. This is significant as some studies compare CCM to one single test, which is not the gold standard in the case of NDS. NCS only measures large fiber function, which is affected later than small nerve fibers in DPN. One study (187), demonstrated that diagnostic ability of CNFL measurement in DM patients is significantly worse if using clinical signs and symptoms as a reference standard in comparison to electrophysiology, plus one sign/symptom as per the Toronto consensus guidelines, which highlights the importance of a standardized diagnostic reference (187).

To explore which of the many measurements derived from CCM could best distinguish patients with and without clinical DPN, as part of each study, the same patients were examined using CCM, and all nerve parameters were derived. For each nerve parameters tested, ROC curves were plotted to determine a CCM cut-off point used to distinguish between patients with and without DPN in the diabetic cohort only. A range of cut-off points was studied to identify the best sensitivity/specificity value for diagnosing DPN for each nerve parameter.

CNFL was the most commonly reported nerve parameter for these studies, with all nine assessing its diagnostic ability and finding significant differences between patients with and without DPN. A range of sensitivity values between 59 and 86% was found and a specificity range of 61-84%, depending on the cut-off value used for diagnosis. The earliest of these studies (62), examined patients using a Tomey confoscan CCM. It is well known that these images are of more inferior quality, making it more challenging to identify nerve fibers during analysis. This is likely the explanation for the significantly lower diagnostic threshold value reported in this study compared to the others presented (**Table 5**).

For corneal nerve fiber density (CNFD) six of the cross-sectional studies (**Table 5**) reported a significant reduction in DM patients with DPN compared to both DM patients without DPN and healthy controls (62, 100, 102, 104, 106, 107). These studies reported sensitivity and specificity ranges as 65-82% and 41-79% respectively. A significantly higher cut off point of 39.2 CNFD no/mm^2^ was defined in T2DM patients in the consortium study, resulting in an increased sensitivity value to 69% (104). This may explain why its specificity is the lowest value of only 41%, as a higher cut-off value may create more false-positive results. It is notable that based on their cohort, Scarr et al. (106) defined the lowest thresholds for diagnosis for both CNFD and CNFL out of the studies using the Heidelberg retinal tomograph (HRT) (III) CCM. This is likely due to their significantly older-aged cohort compared to the other cross-sectional studies as CNFD and CNFL have been shown to reduce with age (188).

For corneal nerve branch density (CNBD), six cross-sectional studies (62, 100, 102, 104, 106, 107) reported a significant reduction in DM with DPN than without DPN. For diagnostic value, the sensitivity (17-100%) (62, 102) and specificity (45-96%) (62, 102) values were significantly more varied, suggesting that this parameter has shown the least promise for DPN diagnosis until now.

There are several strengths to each of the cross-sectional studies. Three used profile-matched healthy controls (101, 102, 106), meaning that differences in measurements between the two groups due to age should have been accounted for, giving a better representation of changes that have occurred due to DPN. Ahmed et al. (101) also looked at the option of combining two corneal nerve parameters for the identification of neuropathy. Two of the studies looked at both manual, and automated software for DPN diagnosis (102, 106) which is significant as automated software for analysis would be required if CCM were to be introduced in large-scale screening.

Perkins et al. (104) in a consortium multi-center study funded by NIH assessed data from a large cohort of 998 subjects. This large cohort of different ethnicities and T1DM and T2DM gave a more accurate representation of the population of people with diabetes instead of focusing on one specific sub-group. Another strength of this NIH funded study was that it suggested an alternative approach of using one lower value chosen to more confidently rule in the presence of neuropathy (maximize specificity) and one higher value determined to simultaneously, more confidently rule out the presence of neuropathy (maximize sensitivity). This combination of decision criteria aims to minimize false positive and negative results. The study found that using this criterion increased their sensitivity to 88% and specificity to 89% using manual methods of analysis. However, this method caused 57.8% of the subjects to be unclassified as they fell between the two limits.

There were several limitations to these cross-sectional studies. Some did not match the clinical profiles of their patients to the control subjects. For example, the patients’ group in Alam et al. (100) being significantly older, with significantly longer disease duration than the T2DM group without neuropathy. Another limitation of two of these studies (101, 106) was that only 1 image per eye was used for analysis. One criterion for choosing this image in the Ahmed et al. (101) study was the most nervous frame. Using this method to choose 1 image per eye instead of calculating an average of 3 images or more may be less time consuming for analysis; however, it is likely to give the false elevation of measurements per patient instead of representing an accurate average.

Another significant issue with these studies is that most of them use the Toronto consensus as to the diagnostic criteria for DPN (100–102, 104, 106), i.e. one abnormal finding as part of NCS, in combination with a symptom or sign of neuropathy (41). As mentioned previously, NCS assesses large fiber function whereas CCM assesses small fiber function.

Despite the variation in results and limitations of the studies, these findings supported the expanded role of CCM in the assessment of diagnosis DPN as a supplement to the vast array of neurological tests currently in use.

### Early Detection of Neuropathy

As there are currently no therapeutic agents approved for DPN treatment, early detection is essential to modify any risk factors. Several studies have specifically investigated CCM findings in early stages of DM and mild levels of DPN.

The published baseline characteristics of T1DM patients as part of the LANDMark study (189) were that corneal nerve fiber length was reduced in patients without clinical neuropathy, based on the Toronto criteria. Another paper written from the same study (190) assessed the use of CCM for distinguishing between control patients and DM patients (156 T1DM, 75 T2DM) with and without clinical DPN. For the patients with DPN, all cases were defined as mild (as defined by QST plus neurophysiology). This study reported a significant reduction in CNFL when comparing patients with and without mild neuropathy, suggesting that CNFL changes may occur early in the course of the disease.

One study (191) assessed the corneal sub-basal plexus in patients with recently diagnosed T2DM (mean duration 2.1± 1.6 years). This study reported significant differences between CNFD, CNBD and CNFL parameters when comparing the patient cohort to the control group, with CNFD emerging as the most sensitive in detecting corneal nerve pathology; indeed 21% of the patients fell below the 2.5^th^ percentile of the control group. For this study, high-adapted software produced an image composed of an image stack. It reconstructed a combined mosaic image with an expanded field of view compared to standard imaging using CCM. This software is also able to correct for artefacts. As this method is not widely used, there is no direct comparison to other studies. To our knowledge, no other studies are assessing recently diagnosed patients with DM (<2 years duration). It must also be considered that in this study, even though patients were diagnosed newly, there may have been a delay in diagnosis, which could have varied between patients.

Another study assessing early nerve changes assessed patients with impaired glucose tolerance (IGT) (192). This study reported evidence that CCM may detect changes in nerve parameters before established diabetes. They reported that in patients with IGT, CNFD and CNBD were significantly reduced with 40.5% of subjects with IGT having significant small-fiber damage based on CNFD reduction compared to control subjects. This agreed with a decrease in IENFD and significantly higher warm thresholds and vibration perception thresholds in the same cohort (192).

### Langerhans Cells in DPN

**Figure 2:**
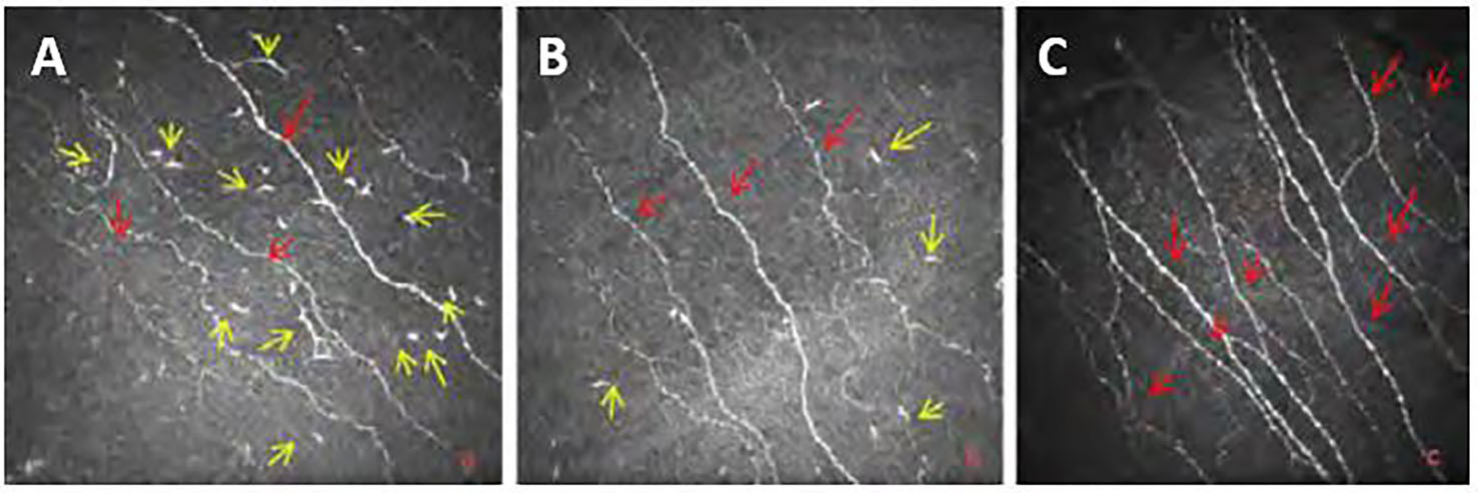
Images from Bowman’s layer of the cornea at **(A)** T2DM with mild neuropathy; **(B)** moderate neuropathy; **(C)** Healthy Control Subject (yellow arrows show LCs and red arrows indicates main corneal nerve c nerve fibers).

The dominant antigen-presenting cells in the cornea and ocular surfaces are Langerhans cells (LCs) and Dendritic cells (DCs) which are derived from the bone marrow and can stimulate both primary and secondary T and B-cell responses (169). It has been shown that Corneal confocal microscopy provides a non-invasive means to readily demonstrate Langerhans cells (LCs) in the cornea of healthy subjects and a range of inflammatory ophthalmic conditions (**Figure 2**) (174, 193). Some studies demonstrated that the number of LCs increases in Diabetic Neuropathy (177, 194–196); however, the LCs activation mechanism is still unclear.

Zhivov et al. (176) assessed the corneal basal epithelial layer and the sub-basal nerve plexus for the presence of LCs in healthy subjects and found that 31% of subjects had LCs present. Tavakoli and colleagues (177) were the first to assess Langerhans cell density with differing severities of diabetic neuropathy (based on NDS scoring compared to controls). This study found a significant increase in the proportion of individuals with LCs in patients with T1DM and T2DM (73.8%) compared to control subjects (46.1%). The study also found that LC density was significantly increased in the patients with diabetes (17.73 ± 1.45) compared to control subjects (6.94 ± 1.58). However, with progression of neuropathy, patients with moderate and severe neuropathy showed a reduction in the LC density in comparison to patients with mild neuropathy and were not significantly different from control subjects. This may suggest that LCs have a role in the early phase of nerve damage. This study only focused on Bowman’s layer which has been shown to have a lower density of LCs in comparison to the epithelial layer (197), so is not an accurate representation of overall LC density in the central cornea. Another limitation of the study was that the Tomey Confoscan CCM was used for imaging which has been shown to underestimate LCs density compared to newer the Heidelberg HRT III CCM (176) and cannot differentiate mature from immature LCs (176).

A more recent study, used the HRT (III) CCM to assess the density of LCs in a cohort of children and adolescents with diabetes and found a higher percentage of patients (85.9%) and controls (69.1%) with LCs present when compared to the previous two studies (176, 177). This study was also able to distinguish between mature and immature cells by classing LCs of less than 50 µm in length, without dendritic structures as immature cells and those greater than 50 µm with dendritic structures were considered as mature cells. A significant increase in mature and immature cells was found, and a correlation existed between CNFD and LCs density (198). However, this study only assessed a specific age-group of the diabetic cohort, so it does not represent the whole diabetic population. Overall, studies investigating LCs density in patients with diabetes are still limited, and more information is required to conclude the effect of diabetes on LCs.

### Comparing CCM and IENFD

**Figure 3:**
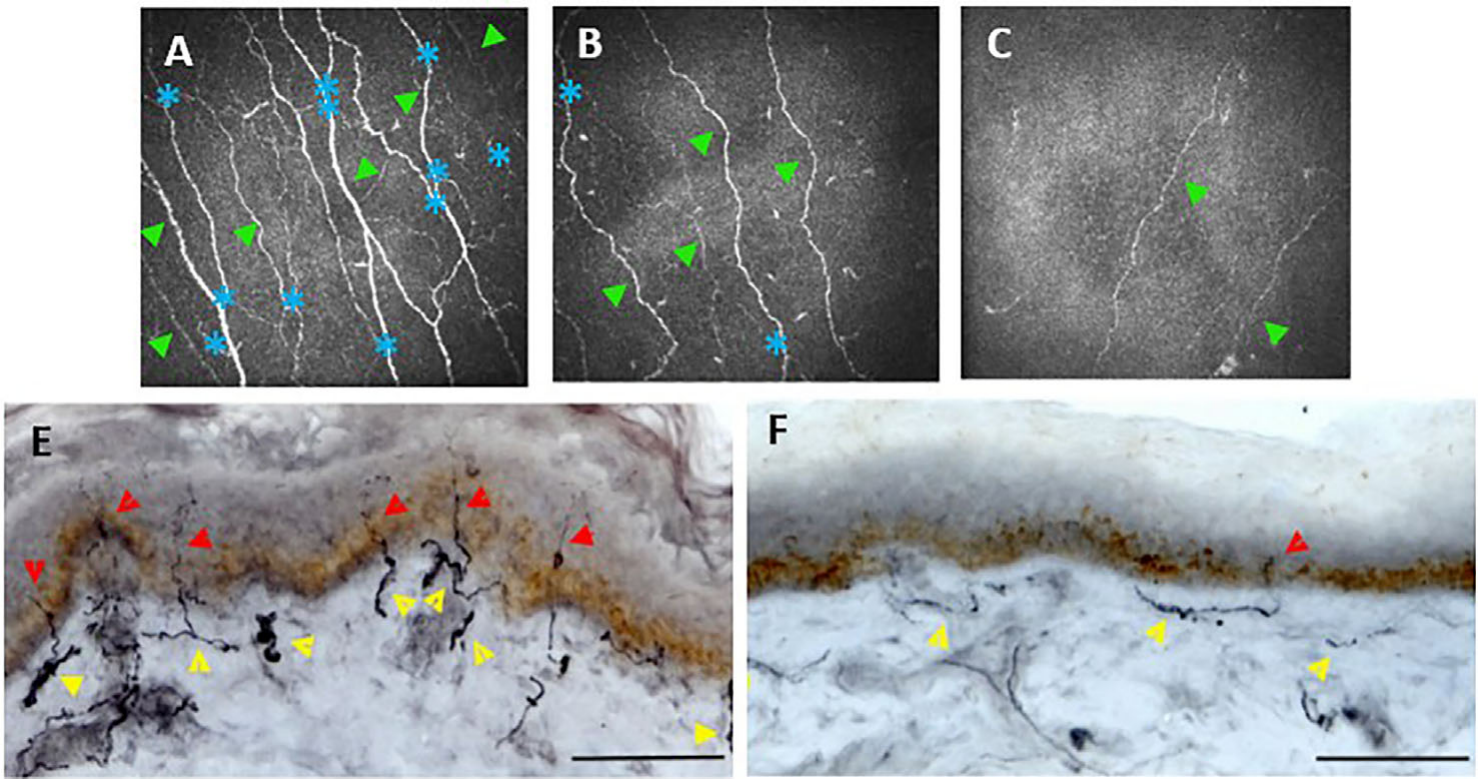
Corneal confocal microscopy images of the corneal, sub-basal nerves **(A–C)**. Healthy control **(A)** shows numerous corneal main nerve fibers (green arrowheads) with branching nerves (blue asterisks). CCM images of patients with diabetes and mild **(B)** or severe **(C)** neuropathy demonstrate reduced corneal nerves and branches. Skin biopsies **(E, F)** immunostained. Healthy control **(E)** shows numerous intraepidermal nerve fibers (red arrowheads) with a well-developed subepidermal nerve plexus (yellow arrowheads). A diabetic patient **(F)** demonstrates reduced subepidermal and minimal intraepidermal nerve fibers. Scale bar = 100 mm. **(E, F)** adapted from (186).

Studies have found CCM to be comparable with measures of IENFD from biopsies in their diagnostic performance for detecting patients with clinical levels of DPN (100, 102) (**Figure 3**). Both studies found no significant difference in their diagnostic efficacy in patients with T1DM.

An older study using the Tomoscan confocal microscope (110) also concluded that both IENFD and CCM assessment accurately quantify small nerve fiber damage in patients with diabetes. Intraepidermal and corneal nerve fiber lengths were also both further reduced in patients with painful compared with painless diabetic neuropathy.

In comparison, one study’s findings, using HRT (III) CCM were notably different (191). This study reported that CCM and IENFD were both able to detect nerve fiber loss in recently diagnosed type 2 diabetes, but mainly in different patients. They, therefore, suggested a possible patchy manifestation pattern of small fiber neuropathy. Only 2.7% of the patients had both abnormal CNFD and IENFD. Abnormal CCM with normal IEND was noted in 20.5% of the diabetic group and 11.0% for vice versa. No correlation between the CCM measures and IENFD was observed. There are possible explanations for these contradictory findings. Firstly, the cohort of patients in this study was all patients with T2DM, all of who had been newly diagnosed (known diabetes duration of ≤1 year). The disease duration was significantly less than that of Chen et al. (102) (DPN+ 39 ± 14 DPN- 23 ± 15 years) and Alam et al. (100) (DSPN+ 37.2 ± 13.1 DSPN- 17.2 ± 12.0 years). These two studies also used comparisons between patients with and without clinical DPN to compare IENFD and CCM, whereas Ziegler et al. (191) only compared patients with T2DM to healthy controls. Lastly, Ziegler et al. used a different location for the IENFD biopsy. This was taken from the lateral calf in comparison to the dorsum of the foot. This more proximal site may have been at less risk IENFD changes or may present a different pattern of loss, as DSPN is known to follow a distal-proximal course.

One issue with the comparison of IENFD with analysis of the corneal sub-basal nerve plexus is that intra-epidermal nerves consist of both unmyelinated C-fibers (90%) and myelinated A-delta fibers (10%) (199), which are both included in the measurement for IENFD. In contrast, the sub-basal nerve plexus is made up of C-fibers only. This means that a direct comparison cannot be made between the two measurements. Although the A-delta fibers only make up 10% of the total number in the epidermal layer, they may be affected differently in DPN than the unmyelinated C-fibers, affecting the overall results.

### Longitudinal Studies for Application of CCM for Assessment DPN

Longitudinal studies suggest that CCM has good predictive value for subsequent DPN (187, 200). Longitudinal analysis of a T1DM cohort showed a mean 1-year change in CNFL was -1.6% in patients with unstable T1DM, while healthy volunteers showed a 5% increase per year (200).

As part of a 4-year follow up study, a study (103) (**Table 5**) found that three corneal nerve parameters were all significant predictors for the development of DPN, with a baseline CNFL of <14.9mm/mm^2^ being the strongest single predictor when compared to 11 other small and large fiber tests. Other studies (105, 201) also reported an association between lower baseline CNFL and DPN development. Pritchard et al. (105) (**Table 5**) found a significant association with longer diabetes duration, higher triglycerides, worsening retinopathy and nephropathy, impaired sensation to temperature and vibration and slower peroneal and sural nerve conduction velocities. However, studies with larger cohorts and patients with type 2 diabetes are needed to confirm these studies’ findings and a more extended period of monitoring. Studies should also ensure a set number of follow-ups over a set period as for Lovblom et al. (103) more than half of the patients had just one follow up visit, meaning that true progression is statistically challenging to prove.

Another prospective study specifically looked at a group of patients with IGT (202). They found that in subjects with IGT, lower baseline CNFD, CNBD, CNFL, and lower mean dendritic length of IENF were the strongest predictors of progression to T2DM over three years. Although significance was not recorded, there appeared to be very similar baseline HbA1c measures between those patients who remained IGT vs those developing T2DM over the three years follow up (42.8 ± 1.2 and 42.4 ± 1.0 respectively(mmol/mol), suggesting that corneal nerve parameters may have been stronger predictors of conversion to T2DM in comparison to baseline HbA1c. Those subjects who returned to normoglycemia showed a significant improvement in their CCM parameters while IENF length continued to decline during the same period. These findings may suggest that corneal nerve fibers regenerate quicker than IENF when glycemic control is improved.

Another observational follow up study (203), examined a small cohort of patients with diabetes (15 T1DM and 10 T2DM) at baseline and follow-up at two years. At follow up, an improvement in glycemic control, cholesterol levels and blood pressure were found and increased CNFD, with a significant correlation between a decrease in HbA1c and CNFD. This demonstrated that improvements in HbA1c might lead to morphological repair of corneal nerve fibers, however, due to the small sample size and mixing of T1DM and T2DM in analysis, it is unclear if these differences are occurring in both types. It must also be noted that this was not planned as an interventional study, meaning there were no placebo controls or randomization, which would need to take place to confirm or reject these findings.

CCM has been used to investigate the sub-basal nerve plexus changes in patients with T1DM post-simultaneous pancreas and Kidney (SPK) transplant. Tavakoli et al. (186) assessed 15 patients at 6 and 12 months SPK transplant and found a significant improvement in all CCM parameters at 12 months. Symptoms, neurophysiology, quantitative sensory testing and skin biopsy results remained unchanged in the same patients. A similar, earlier study using an older CCM model also reported similar findings, with CNFD and CNFL increasing significantly after just six months (204). These studies may demonstrate that CCM can provide a novel non-invasive means to evidence early nerve repair missed by currently advocated assessment techniques. However, an alternative interpretation of this data could be that corneal nerves respond well to the restoration of insulin and normoglycemia. In contrast, other peripheral nerves do not; therefore, CCM may be measuring something unique that is not an accurate biomarker of the condition of peripheral nerves.

### CCM Application in Clinical Trials

Several DPN intervention trials have focused on large fiber function and have generally had ineffective outcomes. More recently, some studies have instead focused on CCM measures as markers for clinical trials of potential new treatments. In a recent pilot trial of seal oil omega-3, polyunsaturated fatty acid supplementation in patients with type 1 diabetes (disease duration 27 ± 14 years) over 12 months (205), there was a significant increase (30.4%) in corneal nerve fiber length, with no change found in NCS velocity or sensory function. Those subjects at high risk for future DPN and those with already diagnosed DPN (as determined by a Toronto clinical neuropathy score of ≥ 1) showed the best treatment response. This study was a single-arm, open-label, proof of concept trial; therefore, no placebo group was used, which is necessary to reduce a trial’s bias.

Another study to determine whether the peptide, ARA 290, improves metabolic control and neuropathic pain in patients with type 2 diabetes used CCM measurements as a co-primary endpoint. This study found that ARA 290 treatment was associated with an increase in corneal nerve fiber density correlated with changes in neuropathic symptoms (206). This study was a double-blind, placebo-controlled investigator-initiated phase II clinical trial whose inclusion criteria were patients with T2DM who also had small fiber neuropathy symptoms. Whether allocation to the treatment and placebo groups was randomized was not discussed in the article. This study’s limitation was that patients assigned to both groups generally had excellent metabolic control (HbA1c = 7.3 ± 0.4 and 6.9 ± 0.2 for treatment and placebo groups respectively), which does not truly represent the clinical population of patients with T2DM. It may be that this treatment is less or more effective for patients with poor metabolic control, comparatively. Finally, disease duration was also not mentioned, so it was unclear if there was a significant difference between the two groups.

These trials may be evidence that, like small fiber damage occurring before large fiber damage, small fibers are also the first to start regenerating after damage. Trials over a longer period, including other small fiber neuropathy measures, are required before these findings can be confirmed.

## Conclusions

There is an un-met need for a simple, reliable and accurate test for the early detection of diabetic peripheral neuropathy (DPN) which may help reduce the incidence of ulcers and amputations in people with diabetes which remains at an all-time high and increases by between 25-20% per annum. Current tests for DPN in primary care require HCPs to conduct and detect late neuropathy. Many people with diabetes do not have an annual foot check despite it being one of the most important of the 8 care processes mandated by NICE. In fact over 500,000 people in England alone never have an annual diabetes related foot examination. This places them at risk of having a first presentation with an active foot ulcer in A&E. Early diagnosis of DPN is critical since early damage to the small nerve fibers in the feet of people with diabetes can be stopped from progressing and even reversed while late signs of DPN and in particular lack of vital protective sensation in the feet cannot be reversed. Although a common and much feared complication, over 30% of people with diabetes remain unaware.

Diabetes UK consistently state that 80% of diabetes related foot complications are preventable yet no practical solution to this huge problem has been proposed. The only primary care test recommended by the National Institute of Health and Care Excellence (NICE) is the 10g Semmes Weinstein monofilament examination (SWME) which is a crude, inaccurate and subjective test for lack of sensation in the feet of people with diabetes - defined as ‘late neuropathy’ which cannot be reversed. Its evidence base is poor and outside NICE it is regarded as a particularly poor test for DPN.

Early diagnosis and timely intervention are thus essential in preventing its development. Whereas measurement of urinary albumin excretion and fundoscopic examinations serve as objective tests for early nephropathy and retinopathy respectively, a comparably objective, accurate test which is unbiased by the patient’s subjective response is lacking for DPN.

Currently advocated diagnostic tests either focus on large nerve fibers, thus are not sensitive to early abnormalities, are too time-consuming and/or are too invasive to be used for repeated measures.

More recently, a number of non-invasive tests have been developed as surrogate measures of DPN. Of these, CCM has shown great potential for the detection of small fiber neuropathy, the earliest manifestation of DPN. CCM has also demonstrated promising prognostic utility and has demonstrated early nerve regeneration post-SPK surgery and as part of several clinical trials.

Given that CCM is a rapid and non-invasive test, it is suitable for large-scale screening for DPN, and advancements in automated analysis software would further improve its promising potential.

In conclusion, there is no optimal biomarker and ideal endpoint available for DPN at the current time. Hence, there is an urgent need to identify the most accurate early biomarker of nerve damage to diagnose DPN in patients’ clinical care better and, in particular, to permit a precise evaluation of future therapies in clinical trials. The global effort among scientists and clinicians, and researchers in the field should address these shortcomings to reduce incidence of complications and to achieve this; the search should continue for better and sensitive tests, screening, and early detection. We need to improve our systematic evaluation of the evidence and promote—from each translational step to the next—the biomarkers with the best evidence and performance at different populations. This will require evaluation of the wider biomarker research agenda. Such evaluation may also benefit more from fostering international collaborations rather than from the fragmented efforts of small, opportunistic studies. “We must learn to measure what we value rather than valuing what we can easily measure”.

## Author Contributions

JC wrote and revised the manuscript. HF, FI, and AS reviewed and revised the paper. MT designed, conceived the study, wrote the major revision and made comments, had full access to all data, and is the guarantor. MT and AS supervised JC. All authors contributed to the article and approved the submitted version.

## Conflict of Interest

The authors declare that the research was conducted in the absence of any commercial or financial relationships that could be construed as a potential conflict of interest.

## Glossary

ACCORD: Action to Control Cardiovascular Risk in Diabetes
ACE: Angiotensin-Converting Enzyme
BFI: Bright Field Immunohistochemistry
BMI: Body Mass Index
CCM: Corneal Confocal Microscopy
CNBD: Corneal Nerve Branch Density
CNFA: Corneal Nerve Fiber Area
CNFD: Corneal Nerve Fiber Density
CNFL: Corneal Nerve Fiber Length
CNFW: Corneal Nerve Fiber Width
CNS: Central Nervous System
CTBD: Corneal Total Branch Density
DAN: Diabetic Autonomic Neuropathy
DN4: Douleur Neuropathique en 4
DNE: Diabetic Neuropathy Examination
DNS: Diabetic Neuropathy Score
DPN: Diabetic Peripheral Neuropathy
DSPN: Distal Symmetrical Polyneuropathy
EDIC: Epidemiology of Diabetes Interventions and Complications
ESC: Electrochemical Skin Conductance
HbA1c: Glycated Hemoglobin
HDL: High-Density Lipoprotein
HES: Hospital Eye Service
HRT: Heidelberg Retinal Tomograph
IENFD: Intra-epidermal Nerve Fiber Density
IF: Indirect Immunofluorescence
IGT: Impaired Glucose Tolerance
IWL: Inferior Whorl Length
LANSS: Leeds Assessment of Neuropathic Symptoms and Signs
LC: Langerhans Cell
LDL: Low-Density Lipoprotein
MNDS: Michigan Neuropathy Disability Score
MNSI: Michigan Neuropathy Screening Instrument
MRI: Magnetic Resonance Imaging
MTHFR: Methylenetetrahydrofolate Reductase
NCS: Nerve Conduction Studies
NDS: Neuropathy Disability Score
NeuPSIG: International Association for the Study of Pain
NEURODIAB: Diabetic Neuropathy Study Group of the European Association for the Study of Diabetes
NIS-LL: Neuropathy Impairment Score in the Lower Limbs
NPQ: Neuropathic Pain Questionnaire
NPV: Negative Predictive Value
NPSI: Neuropathic Pain Symptom Inventory
NSS: Neuropathy Symptoms Score
QSART: Quantitative Sudomotor Axon Testing
QST: Quantitative Sensory Testing
SFN: Small Fiber Neuropathy
T1DM: Type 1 Diabetes Mellitus
T2DM: Type 2 Diabetes Mellitus
TC: Tortuosity Coefficient
TCNS: Toronto Clinical Neuropathy Score
UENS: Utah Early Neuropathy Scale

## References

[1] Neeland IJ Patel KV. Chapter 4 - Diabetes: Key Markers of Injury and Prognosis. In: Nambi V, editor. Biomarkers in Cardiovascular Disease. Netherlands: Elsevier (2019). p. 41–51.

[2] Internation Diabetes Federation. IDF Diabetes Atlas. Brussels, Belgium: International Diabetes Federation (2019). Available at: https://diabetesatlas.org/en/sections/worldwide-toll-of-diabetes.html.

[3] Diabetes UK. Us, diabetes and a lot of facts and stats. London, UK: Diabetes UK (2019). Available at: https://www.diabetes.org.uk/resources-s3/2019-02/1362B_Facts%20and%20stats%20Update%20Jan%202019_LOW%20RES_EXTERNAL.pdf.

[4] Hicks CW Selvin E. Epidemiology of Peripheral Neuropathy and Lower Extremity Disease in Diabetes. Curr Diabetes Rep (2019) 19(10):86. 10.1007/s11892-019-1212-8 PMC6755905 31456118

[5] Pop-Busui R Boulton AJM Feldman EL Bril V Freeman R Malik RA. Diabetic Neuropathy: A Position Statement by the American Diabetes Association. Diabetes Care (2017) 40(1):136. 10.2337/dc16-2042 27999003 PMC6977405

[6] Sloan G Shillo P Selvarajah D Wu J Wilkinson ID Tracey I. A new look at painful diabetic neuropathy. Diabetes Res Clin Pract (2018) 144:177–91. 10.1016/j.diabres.2018.08.020 30201394

[7] Albers JW Herman WH Pop-Busui R Feldman EL Martin CL Cleary PA. Effect of prior intensive insulin treatment during the Diabetes Control and Complications Trial (DCCT) on peripheral neuropathy in type 1 diabetes during the Epidemiology of Diabetes Interventions and Complications (EDIC) Study. Diabetes Care (2010) 33(5):1090–6. 10.2337/dc09-1941 PMC2858182 20150297

[8] Kioskli K Scott W Winkley K Kylakos S McCracken LM. Psychosocial Factors in Painful Diabetic Neuropathy: A Systematic Review of Treatment Trials and Survey Studies. Pain Med (2019) 20(9):1756–73. 10.1093/pm/pnz071 30980660

[9] Singh N Armstrong DG Lipsky BA. Preventing foot ulcers in patients with diabetes. Jama (2005) 293(2):217–28. 10.1001/jama.293.2.217 15644549

[10] Ndip A Ebah L Mbako A. Neuropathic diabetic foot ulcers - evidence-to-practice. Int J Gen Med (2012) 5:129–34. 10.2147/IJGM.S10328 PMC3282596 22371655

[11] Smith BE. Chapter 3 - Focal and entrapment neuropathies. In: Zochodne DW, editor. Handbook of Clinical Neurology, vol. 126. Amsterdam, Netherlands: Elsevier (2014). p. 31–43. 10.1016/B978-0-444-53480-4.00003-5 25410212

[12] National Institute for Health and Care Excellence (NICE). Diabetic foot problems: prevention and management. London, UK: NICE (2015). Available at: https://www.nice.org.uk/guidance/ng19. 26741017

[13] Thorud JC Plemmons B Buckley CJ Shibuya N Jupiter DC. Mortality After Nontraumatic Major Amputation Among Patients With Diabetes and Peripheral Vascular Disease: A Systematic Review. J Foot Ankle Surg (2016) 55(3):591–9. 10.1053/j.jfas.2016.01.012 26898398

[14] Manzano GM Giuliano LMP Nóbrega JAM. A brief historical note on the classification of nerve fibers. Arquivos Neuro Psiquiatria (2008) 66:117–9. 10.1590/S0004-282X2008000100033 18392435

[15] Gregg EW Gu Q Williams D de Rekeneire N Cheng YJ Geiss L. Prevalence of lower extremity diseases associated with normal glucose levels, impaired fasting glucose, and diabetes among U.S. adults aged 40 or older. Diabetes Res Clin Pract (2007) 77(3):485–8. 10.1016/j.diabres.2007.01.005 17306411

[16] Tesfaye S Chaturvedi N Eaton SE Ward JD Manes C Ionescu-Tirgoviste C. Vascular risk factors and diabetic neuropathy. N Engl J Med (2005) 352(4):341–50. 10.1056/NEJMoa032782 15673800

[17] Martin CL Albers JW Pop-Busui R. Neuropathy and Related Findings in the Diabetes Control and Complications Trial/Epidemiology of Diabetes Interventions and Complications Study. Diabetes Care (2014) 37(1):31–8. 10.2337/dc13-2114 PMC3868000 24356595

[18] Trotta D Verrotti A Salladini C Chiarelli F. Diabetic neuropathy in children and adolescents. Pediatr Diabetes (2004) 5(1):44–57. 10.1111/j.1399-543X.2004.00041.x 15043690

[19] Solders G Thalme B Aguirre-Aquino M Brandt L Berg U Persson A. Nerve conduction and autonomic nerve function in diabetic children. A 10-year follow-up study. Acta Paediatrica (1997) 86(4):361–6. 10.1111/j.1651-2227.1997.tb09023.x 9174220

[20] Ismail-Beigi F Craven T Banerji MA Basile J Calles J Cohen RM. Effect of intensive treatment of hyperglycaemia on microvascular outcomes in type 2 diabetes: an analysis of the ACCORD randomised trial. Lancet (2010) 376(9739):419–30. 10.1016/S0140-6736(10)60576-4 PMC4123233 20594588

[21] Duckworth W Abraira C Moritz T Reda D Emanuele N Reaven PD. Glucose control and vascular complications in veterans with type 2 diabetes. N Engl J Med (2009) 360(2):129–39. 10.1056/NEJMoa0808431 19092145

[22] Jende JME Groener JB Oikonomou D Heiland S Kopf S Pham M. Diabetic neuropathy differs between type 1 and type 2 diabetes: Insights from magnetic resonance neurography. Ann Neurol (2018) 83(3):588–98. 10.1002/ana.25182 29443416

[23] Andersen ST Witte DR Dalsgaard EM Andersen H Nawroth P Fleming T. Risk Factors for Incident Diabetic Polyneuropathy in a Cohort With Screen-Detected Type 2 Diabetes Followed for 13 Years: ADDITION-Denmark. Diabetes Care (2018) 41(5):1068–75. 10.2337/dc17-2062 29487078

[24] Feldman EL Callaghan BC Pop-Busui R Zochodne DW Wright DE Bennett DL. Diabetic neuropathy. Nat Rev Dis Primers (2019) 5(1):41. 10.1038/s41572-019-0092-1 31197153

[25] Tesfaye S Stevens LK Stephenson JM Fuller JH Plater M Ionescu-Tirgoviste C. Prevalence of diabetic peripheral neuropathy and its relation to glycaemic control and potential risk factors: the EURODIAB IDDM Complications Study. Diabetologia (1996) 39(11):1377–84. 10.1007/s001250050586 8933008

[26] Abbott CA Garrow AP Carrington AL Morris J Van Ross ER Boulton AJ. Foot Ulcer Risk Is Lower in South-Asian and African-Caribbean Compared With European Diabetic Patients in the U.K. Diabetes Care (2005) 28(8):1869–75. 10.2337/diacare.28.8.1869 16043725

[27] Fadavi H Tavakoli M Foden P Ferdousi M Petropoulos IN Jeziorska M. Explanations for less small fibre neuropathy in South Asian versus European subjects with type 2 diabetes in the UK. Diabetes/Metab Res Rev (2018) 34(7):e3044. 10.1002/dmrr.3044 PMC6220759 29972725

[28] Tahrani AA Altaf QA Piya MK Barnett AH. Peripheral and Autonomic Neuropathy in South Asians and White Caucasians with Type 2 Diabetes Mellitus: Possible Explanations for Epidemiological Differences. J Diabetes Res (2017) 2017:1273789. 10.1155/2017/1273789 28409160 PMC5376938

[29] Zhou Z Sun B Huang S Zhu C Bian M. Glycemic variability: adverse clinical outcomes and how to improve it? Cardiovasc Diabetol (2020) 19(1):102. 10.1186/s12933-020-01085-6 32622354 PMC7335439

[30] Hirsch IB. Glycemic Variability and Diabetes Complications: Does It Matter? Of Course It Does! Diabetes Care (2015) 38(8):1610–14. 10.2337/dc14-2898 26207054

[31] Gaede P Lund-Andersen H Parving HH Pedersen O. Effect of a multifactorial intervention on mortality in type 2 diabetes. N Engl J Med (2008) 358(6):580–91. 10.1056/NEJMoa0706245 18256393

[32] Ziegler D Behler M Schroers-Teuber M Roden M. Near-normoglycaemia and development of neuropathy: a 24-year prospective study from diagnosis of type 1 diabetes. BMJ Open (2015) 5(6):e006559. 10.1136/bmjopen-2014-006559 PMC4479996 26109108

[33] Kluding PM Pasnoor M Singh R Jernigan S Farmer K Rucker J. The effect of exercise on neuropathic symptoms, nerve function, and cutaneous innervation in people with diabetic peripheral neuropathy. J Diabetes Complications (2012) 26(5):424–9. 10.1016/j.jdiacomp.2012.05.007 PMC3436981 22717465

[34] Smith AG Russell J Feldman EL Goldstein J Peltier A Smith S. Lifestyle Intervention for Pre-Diabetic Neuropathy. Diabetes Care (2006) 29(6):1294–9. 10.2337/dc06-0224 16732011

[35] Ishibashi F Taniguchi M Kosaka A Uetake H Tavakoli M. Improvement in Neuropathy Outcomes With Normalizing HbA1c in Patients With Type 2 Diabetes. Diabetes Care (2018) 42(1):110–8. 10.2337/dc18-1560 30455338

[36] Cardoso CRL Leite NC Moram CBM Salles GF. Long-term visit-to-visit glycemic variability as predictor of micro- and macrovascular complications in patients with type 2 diabetes: The Rio de Janeiro Type 2 Diabetes Cohort Study. Cardiovasc Diabetol (2018) 17(1):33. 10.1186/s12933-018-0677-0 29477146 PMC6389075

[37] Pinto MV Rosa L Pinto LF Dantas JR Salles GF Zajdenverg L. HbA1c variability and long-term glycemic control are linked to peripheral neuropathy in patients with type 1 diabetes. Diabetol Metab Syndr (2020) 12:85. 10.1186/s13098-020-00594-4 33042229 PMC7539505

[38] American Diabetes Association. 11. Microvascular Complications and Foot Care: Standards of Medical Care in Diabetes-2020. Diabetes Care (2020) 43(Suppl 1):S135–s51. 10.2337/dc20-S011 31862754

[39] Bakker K Apelqvist J Schaper NC. Practical guidelines on the management and prevention of the diabetic foot 2011. Diabetes Metab Res Rev (2012) 28 (Suppl 1):225–31. 10.1002/dmrr.2253 22271742

[40] Yang Z Zhang Y Chen R Huang Y Ji L Sun F. Simple tests to screen for diabetic peripheral neuropathy. Cochrane Database Syst Rev (2018) 7:CD010975. 10.1002/14651858.CD010975.pub2

[41] Tesfaye S Boulton AJM Dyck PJ Freeman R Horowitz M Kempler P. Diabetic neuropathies: update on definitions, diagnostic criteria, estimation of severity, and treatments. Diabetes Care (2010) 33(10):2285–93. 10.2337/dc10-1303 PMC2945176 20876709

[42] Feng Y Schlösser FJ Sumpio BE. The Semmes Weinstein monofilament examination as a screening tool for diabetic peripheral neuropathy. J Vasc Surg (2009) 50(3):675–82. 10.1016/j.jvs.2009.05.017 19595541

[43] Dros J Wewerinke A Bindels PJ van Weert HC. Accuracy of monofilament testing to diagnose peripheral neuropathy: a systematic review. Ann Fam Med (2009) 7(6):555–8. 10.1370/afm.1016 PMC2775618 19901316

[44] Petropoulos IN Ponirakis G Khan A Almuhannadi H Gad H Malik RA. Diagnosing Diabetic Neuropathy: Something Old, Something New. Diabetes Metab J (2018) 42(4):255–69. 10.4093/dmj.2018.0056 PMC6107364 30136449

[45] Meijer JW Smit AJ Sonderen EV Groothoff JW Eisma WH Links TP. Symptom scoring systems to diagnose distal polyneuropathy in diabetes: the Diabetic Neuropathy Symptom score. Diabetes Med (2002) 19(11):962–5. 10.1046/j.1464-5491.2002.00819.x 12421436

[46] Young MJ Boulton AJ MacLeod AF Williams DR Sonksen PH. A multicentre study of the prevalence of diabetic peripheral neuropathy in the United Kingdom hospital clinic population. Diabetologia (1993) 36(2):150–4. 10.1007/BF00400697 8458529

[47] Zilliox LA Ruby SK Singh S Zhan M Russell JW. Clinical neuropathy scales in neuropathy associated with impaired glucose tolerance. J Diabetes Complications (2015) 29(3):372–7. 10.1016/j.jdiacomp.2015.01.011 PMC4558101 25690405

[48] Sharma S Kerry C Atkins H Rayman G. The Ipswich Touch Test: a simple and novel method to screen patients with diabetes at home for increased risk of foot ulceration. Diabetes Med (2014) 31(9):1100–3. 10.1111/dme.12450 24673517

[49] Rayman G Vas PR Baker N Taylor CG Jr Gooday C Alder AI. The Ipswich Touch Test: a simple and novel method to identify inpatients with diabetes at risk of foot ulceration. Diabetes Care (2011) 34(7):1517–8. 10.2337/dc11-0156 PMC3120164 21593300

[50] Zaslansky R Yarnitsky D. Clinical applications of quantitative sensory testing (QST). J Neurol Sci (1998) 153(2):215–38. 10.1016/S0022-510X(97)00293-1 9511880

[51] Themistocleous AC Ramirez JD Serra J Bennett DL. The clinical approach to small fibre neuropathy and painful channelopathy. Pract Neurol (2014) 14(6):368–79. 10.1136/practneurol-2013-000758 PMC4251302 24778270

[52] Lin Y-H Hsieh S-C Chao C-C Chang Y-C Hsieh S-T. Influence of aging on thermal and vibratory thresholds of quantitative sensory testing. J Peripheral Nervous System (2005) 10(3):269–81. 10.1111/j.1085-9489.2005.10305.x 16221286

[53] Lee JA Halpern EM Lovblom LE Yeung E Bril V Perkins BA. Reliability and validity of a point-of-care sural nerve conduction device for identification of diabetic neuropathy. PloS One (2014) 9(1):e86515. 10.1371/journal.pone.0086515 24466129 PMC3899274

[54] Perkins BA Grewal J Ng E Ngo M Bril V. Validation of a Novel Point-of-Care Nerve Conduction Device for the Detection of Diabetic Sensorimotor Polyneuropathy. Diabetes Care (2006) 29(9):2023. 10.2337/dc08-0500 16936147

[55] Killian JM Foreman PJ. Clinical utility of dorsal sural nerve conduction studies. Muscle Nerve (2001) 24(6):817–20. 10.1002/mus.1074 11360266

[56] Shabeeb D Najafi M Hasanzadeh G Hadian MR Musa AE Shirazi A. Electrophysiological measurements of diabetic peripheral neuropathy: A systematic review. Diabetes Metab Syndrome: Clin Res Rev (2018) 12(4):591–600. 10.1016/j.dsx.2018.03.026 29610062

[57] Bril V. Role of Electrophysiological Studies in Diabetic Neuropathy. Can J Neurol Sci/J Canadien Des Sci Neurologiques (1994) 21(S4):S8–S12. 10.1017/S0317167100040683 7874611

[58] Dyck PJ Overland CJ Low PA Litchy WJ Davies JL Dyck PJB. Signs and symptoms versus nerve conduction studies to diagnose diabetic sensorimotor polyneuropathy: Cl vs. NPhys trial. Muscle Nerve (2010) 42(2):157–64. 10.1002/mus.21661 PMC2956592 20658599

[59] Ragé M Van Acker N Knaapen MW Timmers M Streffer J Hermans MP. Asymptomatic small fiber neuropathy in diabetes mellitus: investigations with intraepidermal nerve fiber density, quantitative sensory testing and laser-evoked potentials. J Neurol (2011) 258(10):1852–64. 10.1007/s00415-011-6031-z 21472496

[60] Løseth S Stålberg E Jorde R Mellgren SI. Early diabetic neuropathy: thermal thresholds and intraepidermal nerve fibre density in patients with normal nerve conduction studies. J Neurol (2008) 255(8):1197–202. 10.1007/s00415-008-0872-0 18574618

[61] Ostrovski I Lovblom LE Farooqi MA Scarr D Boulet G Hertz P. Reproducibility of In Vivo Corneal Confocal Microscopy Using an Automated Analysis Program for Detection of Diabetic Sensorimotor Polyneuropathy. PloS One (2015) 10(11):e0142309. 10.1371/journal.pone.0142309 26539984 PMC4634969

[62] Tavakoli M Quattrini C Abbott C Kallinikos P Marshall A Finnigan J. Corneal confocal microscopy: a novel noninvasive test to diagnose and stratify the severity of human diabetic neuropathy. Diabetes Care (2010) 33(8):1792–7. 10.2337/dc10-0253 PMC2909064 20435796

[63] Tavakoli M Malik RA. Corneal confocal microscopy: a novel non-invasive technique to quantify small fibre pathology in peripheral neuropathies. J Visualized Experiments: JoVE 2011(47):e2194. 10.3791/2194 PMC3182640 21248693

[64] Tentolouris N Achtsidis V Marinou K Katsilambros N. Evaluation of the Self-Administered Indicator Plaster Neuropad for the Diagnosis of Neuropathy in Diabetes. Diabetes Care (2008) 31(2):236. 10.2337/dc07-1942 18025406

[65] Quattrini C Jeziorska M Tavakoli M Begum P Boulton AJM Malik RA. The Neuropad test: a visual indicator test for human diabetic neuropathy. Diabetologia (2008) 51(6):1046–50. 10.1007/s00125-008-0987-y 18368386

[66] Ponirakis G Petropoulos IN Fadavi H Alam U Asghar O Marshall A. The diagnostic accuracy of Neuropad for assessing large and small fibre diabetic neuropathy. Diabetic Med: J Br Diabetic Assoc (2014) 31(12):1673–80. 10.1111/dme.12536 PMC4236278 24975286

[67] Papanas N Papatheodorou K Christakidis D Papazoglou D Giassakis G Piperidou H. Evaluation of a new indicator test for sudomotor function (Neuropad) in the diagnosis of peripheral neuropathy in type 2 diabetic patients. Exp Clin Endocrinol Diabetes (2005) 113(4):195–8. 10.1055/s-2005-837735 15891953

[68] Liatis S Marinou K Tentolouris N Pagoni S Katsilambros N. Usefulness of a new indicator test for the diagnosis of peripheral and autonomic neuropathy in patients with diabetes mellitus. Diabetic Med (2007) 24(12):1375–80. 10.1111/j.1464-5491.2007.02280.x 17941862

[69] Kamenov ZA Petrova JJ Christov VG. Diagnosis of diabetic neuropathy using simple somatic and a new autonomic (neuropad) tests in the clinical practice. Exp Clin Endocrinol Diabetes (2010) 118(4):226–33. 10.1055/s-0030-1247565 20200815

[70] Yajnik CS Kantikar VV Pande AJ Deslypere JP. Quick and simple evaluation of sudomotor function for screening of diabetic neuropathy. ISRN Endocrinol (2012) 2012:103714. 10.5402/2012/103714 22830040 PMC3399356

[71] Smith AG Lessard M Reyna S Doudova M Singleton JR. The diagnostic utility of Sudoscan for distal symmetric peripheral neuropathy. J Diabetes its complications (2014) 28(4):511–6. 10.1016/j.jdiacomp.2014.02.013 PMC4219320 24661818

[72] Selvarajah D Cash T Davies J Sankar A Rao G Grieg M. SUDOSCAN: A Simple, Rapid, and Objective Method with Potential for Screening for Diabetic Peripheral Neuropathy. PloS One (2015) 10(10):e0138224. 10.1371/journal.pone.0138224 26457582 PMC4601729

[73] Krieger S-M Reimann M Haase R Henkel E Hanefeld M Ziemssen T. Sudomotor Testing of Diabetes Polyneuropathy. Front Neurol (2018) 9:803. 10.3389/fneur.2018.00803 30319533 PMC6168653

[74] Casellini CM Parson HK Richardson MS Nevoret ML Vinik AI. Sudoscan, a noninvasive tool for detecting diabetic small fiber neuropathy and autonomic dysfunction. Diabetes Technol Ther (2013) 15(11):948–53. 10.1089/dia.2013.0129 PMC3817891 23889506

[75] Thaisetthawatkul P Fernandes Filho JA Herrmann DN. Contribution of QSART to the diagnosis of small fiber neuropathy. Muscle Nerve (2013) 48(6):883–8. 10.1002/mus.23891 23649502

[76] Buchmann SJ Penzlin AI Kubasch ML Illigens BM Siepmann T. Assessment of sudomotor function. Clin Auton Res (2019) 29(1):41–53. 10.1007/s10286-018-0530-2 29737432

[77] Feldman EL Stevens MJ Thomas PK Brown MB Canal N Greene DA. A practical two-step quantitative clinical and electrophysiological assessment for the diagnosis and staging of diabetic neuropathy. Diabetes Care (1994) 17(11):1281–9. 10.2337/diacare.17.11.1281 7821168

[78] Bennett M. The LANSS Pain Scale: the Leeds assessment of neuropathic symptoms and signs. Pain (2001) 92(1-2):147–57. 10.1016/S0304-3959(00)00482-6 11323136

[79] Bril V. NIS-LL: the primary measurement scale for clinical trial endpoints in diabetic peripheral neuropathy. Eur Neurol (1999) 41(Suppl 1):8–13. 10.1159/000052074 10023123

[80] Bril V Tomioka S Buchanan RA Perkins BA. Group tmS. Reliability and validity of the modified Toronto Clinical Neuropathy Score in diabetic sensorimotor polyneuropathy. Diabetic Med (2009) 26(3):240–6. 10.1111/j.1464-5491.2009.02667.x PMC2871179 19317818

[81] Gupta H Jin KH Nguyen HQ McCann MT Unser M. CNN-Based Projected Gradient Descent for Consistent CT Image Reconstruction. IEEE Trans Med Imaging (2018) 37(6):1440–53. 10.1109/TMI.2018.2832656 29870372

[82] Hawker GA Mian S Kendzerska T French M. Measures of adult pain: Visual Analog Scale for Pain (VAS Pain), Numeric Rating Scale for Pain (NRS Pain), McGill Pain Questionnaire (MPQ), Short-Form McGill Pain Questionnaire (SF-MPQ), Chronic Pain Grade Scale (CPGS), Short Form-36 Bodily Pain Scale (SF-36 BPS), and Measure of Intermittent and Constant Osteoarthritis Pain (ICOAP). Arthritis Care Res (Hoboken) (2011) 63 Suppl 11:S240–52. 10.1002/acr.20543 22588748

[83] Herman WH Pop-Busui R Braffett BH Martin CL Cleary PA Albers JW. Use of the Michigan Neuropathy Screening Instrument as a measure of distal symmetrical peripheral neuropathy in Type 1 diabetes: results from the Diabetes Control and Complications Trial/Epidemiology of Diabetes Interventions and Complications. Diabetes Med (2012) 29(7):937–44. 10.1111/j.1464-5491.2012.03644.x PMC3641573 22417277

[84] Meijer J-WG Bosma E Lefrandt JD Links TP Smit AJ Stewart RE. Clinical Diagnosis of Diabetic Polyneuropathy With the Diabetic Neuropathy Symptom and Diabetic Neuropathy Examination Scores. Diabetes Care (2003) 26(3):697. 10.2337/diacare.26.3.697 12610024

[85] Meijer JW van Sonderen E Blaauwwiekel EE Smit AJ Groothoff JW Eisma WH. Diabetic neuropathy examination: a hierarchical scoring system to diagnose distal polyneuropathy in diabetes. Diabetes Care (2000) 23(6):750–3. 10.2337/diacare.23.6.750 10840990

[86] Spallone V Morganti R D’Amato C Greco C Cacciotti L Marfia GA. Validation of DN4 as a screening tool for neuropathic pain in painful diabetic polyneuropathy. Diabetes Med (2012) 29(5):578–85. 10.1111/j.1464-5491.2011.03500.x 22023377

[87] Asad A Hameed MA Khan UA Ahmed N Butt MU. Reliability of the neurological scores for assessment of sensorimotor neuropathy in type 2 diabetics. J Pak Med Assoc (2010) 60(3):166–70. 20225769

[88] Melzack R Katz J. The McGill Pain Questionnaire: Appraisal and current status. In: Handbook of pain assessment, 2nd ed. New York, NY, US: The Guilford Press (2001). p. 35–52.

[89] Melzack R. The short-form McGill Pain Questionnaire. Pain (1987) 30(2):191–7. 10.1016/0304-3959(87)91074-8 3670870

[90] Dyck PJ Sherman WR Hallcher LM John Service F O’Brien PC Grina LA. Human diabetic endoneurial sorbitol, fructose, and myo-inositol related to sural nerve morphometry. Ann Neurol (1980) 8(6):590–6. 10.1002/ana.410080608 7212646

[91] Abbott CA Carrington AL Ashe H Bath S Every LC Griffiths J. The North-West Diabetes Foot Care Study: incidence of, and risk factors for, new diabetic foot ulceration in a community-based patient cohort. Diabetes Med (2002) 19(5):377–84. 10.1046/j.1464-5491.2002.00698.x 12027925

[92] Bril V Perkins BA. Validation of the Toronto Clinical Scoring System for diabetic polyneuropathy. Diabetes Care (2002) 25(11):2048–52. 10.2337/diacare.25.11.2048 12401755

[93] Moghtaderi A Bakhshipour A Rashidi H. Validation of Michigan neuropathy screening instrument for diabetic peripheral neuropathy. Clin Neurol Neurosurg (2006) 108(5):477–81. 10.1016/j.clineuro.2005.08.003 16150538

[94] Weisman A Bril V Ngo M Lovblom LE Halpern EM Orszag A. Identification and prediction of diabetic sensorimotor polyneuropathy using individual and simple combinations of nerve conduction study parameters. PloS One (2013) 8(3):e58783. 10.1371/journal.pone.0058783 23533591 PMC3606395

[95] Litchy WJ Albers JW Wolfe J Bolton CF Walsh N Klein CJ. Proficiency of nerve conduction using standard methods and reference values (Cl. NPhys Trial 4). Muscle Nerve (2014) 50(6):900–8. 10.1002/mus.24243 PMC4169346 24644133

[96] Wahren J Foyt H Daniels M Arezzo JC. Long-Acting C-Peptide and Neuropathy in Type 1 Diabetes: A 12-Month Clinical Trial. Diabetes Care (2016) 39(4):596–602. 10.2337/dc15-2068 26884473

[97] Ruggenenti P Lauria G Iliev IP Fassi A Ilieva AP Rota S. Effects of manidipine and delapril in hypertensive patients with type 2 diabetes mellitus: the delapril and manidipine for nephroprotection in diabetes (DEMAND) randomized clinical trial. Hypertension (2011) 58(5):776–83. 10.1161/HYPERTENSIONAHA.111.174474 21931073

[98] Dyck PJ Norell JE Tritschler H Schuette K Samigullin R Ziegler D. Challenges in design of multicenter trials: end points assessed longitudinally for change and monotonicity. Diabetes Care (2007) 30(10):2619–25. 10.2337/dc06-2479 17513707

[99] Callaghan BC Burke JF Rodgers A McCammon R Langa KM Feldman EL. Expenditures in the elderly with peripheral neuropathy: Where should we focus cost-control efforts? Neurol Clin Pract (2013) 3(5):421–30. 10.1212/CPJ.0b013e3182a78fb1 PMC3806930 24175158

[100] Alam U Jeziorska M Petropoulos IN Asghar O Fadavi H Ponirakis G. Diagnostic utility of corneal confocal microscopy and intra-epidermal nerve fibre density in diabetic neuropathy. PloS One (2017) 12(7):e0180175. 10.1371/journal.pone.0180175 28719619 PMC5515394

[101] Ahmed A Bril V Orszag A Paulson J Yeung E Ngo M. Detection of diabetic sensorimotor polyneuropathy by corneal confocal microscopy in type 1 diabetes: a concurrent validity study. Diabetes Care (2012) 35(4):821–8. 10.2337/dc11-1396 PMC3308301 22323412

[102] Chen X Graham J Dabbah MA Petropoulos IN Ponirakis G Asghar O. Small nerve fiber quantification in the diagnosis of diabetic sensorimotor polyneuropathy: comparing corneal confocal microscopy with intraepidermal nerve fiber density. Diabetes Care (2015) 38(6):1138–44. 10.2337/dc14-2422 PMC4439535 25795415

[103] Lovblom LE Halpern EM Wu T Kelly D Ahmed A Boulet G. In Vivo Corneal Confocal Microscopy and Prediction of Future-Incident Neuropathy in Type 1 Diabetes: A Preliminary & Longitudinal Analysis. Can J Diabetes (2015) 39(5):390–7. 10.1016/j.jcjd.2015.02.006 25936902

[104] Perkins BA Lovblom LE Bril V Scarr D Ostrovski I Orszag A. Corneal confocal microscopy for identification of diabetic sensorimotor polyneuropathy: a pooled multinational consortium study. Diabetologia (2018) 61(8):1856–61. 10.1007/s00125-018-4653-8 PMC6061173 29869146

[105] Pritchard N Edwards K Russell AW Perkins BA Malik RA Efron N. Corneal Confocal Microscopy Predicts 4-Year Incident Peripheral Neuropathy in Type 1 Diabetes. Diabetes Care (2015) 38(4):671–5. 10.2337/dc14-2114 25573881

[106] Scarr D Lovblom LE Lovshin JA Boulet G Farooqi MA Orszag A. Lower corneal nerve fibre length identifies diabetic neuropathy in older adults with diabetes: results from the Canadian Study of Longevity in Type 1 Diabetes. Diabetologia (2017) 60(12):2529–31. 10.1007/s00125-017-4439-4 28971222

[107] Tavakoli M Begum P McLaughlin J Malik RA. Corneal confocal microscopy for the diagnosis of diabetic autonomic neuropathy. Muscle Nerve (2015) 52(3):363–70. 10.1002/mus.24553 25556884

[108] Sharma S Vas PR Rayman G. Assessment of diabetic neuropathy using a point-of-care nerve conduction device shows significant associations with the LDIFLARE method and clinical neuropathy scoring. J Diabetes Sci Technol (2015) 9(1):123–31. 10.1177/1932296814551044 PMC4495534 25231114

[109] Umapathi T Tan WL Loke SC Soon PC Tavintharan S Chan YH. Intraepidermal nerve fiber density as a marker of early diabetic neuropathy. Muscle Nerve (2007) 35(5):591–8. 10.1002/mus.20732 17221881

[110] Quattrini C Tavakoli M Jeziorska M Kallinikos P Tesfaye S Finnigan J. Surrogate markers of small fiber damage in human diabetic neuropathy. Diabetes (2007) 56(8):2148–54. 10.2337/db07-0285 17513704

[111] Lauria G Hsieh ST Johansson O Kennedy WR Leger JM Mellgren SI. European Federation of Neurological Societies/Peripheral Nerve Society Guideline on the use of skin biopsy in the diagnosis of small fiber neuropathy. Report of a joint task force of the European Federation of Neurological Societies and the Peripheral Nerve Society. Eur J Neurol (2010) 17(7):903–12. 10.1111/j.1468-1331.2010.03023.x 20642627

[112] Lauria G Cornblath DR Johansson O McArthur JC Mellgren SI Nolano M. EFNS guidelines on the use of skin biopsy in the diagnosis of peripheral neuropathy. Eur J Neurol (2005) 12(10):747–58. 10.1111/j.1468-1331.2005.01260.x 16190912

[113] Provitera V Nolano M Stancanelli A Caporaso G Vitale DF Santoro L. Intraepidermal nerve fiber analysis using immunofluorescence with and without confocal microscopy. Muscle Nerve (2015) 51(4):501–4. 10.1002/mus.24338 25043126

[114] Nolano M Biasiotta A Lombardi R Provitera V Stancanelli A Caporaso G. Epidermal innervation morphometry by immunofluorescence and bright-field microscopy. J Peripher Nerv Syst (2015) 20(4):387–91. 10.1111/jns.12146 26309146

[115] Lauria G Bakkers M Schmitz C Lombardi R Penza P Devigili G. Intraepidermal nerve fiber density at the distal leg: a worldwide normative reference study. J Peripheral Nervous System (2010) 15(3):202–7. 10.1111/j.1529-8027.2010.00271.x 21040142

[116] Vlčková-Moravcová E Bednařík J Dušek L Toyka KV Sommer C. Diagnostic validity of epidermal nerve fiber densities in painful sensory neuropathies. Muscle Nerve (2008) 37(1):50–60. 10.1002/mus.20889 17763459

[117] Chien HF Tseng TJ Lin WM Yang CC Chang YC Chen RC. Quantitative pathology of cutaneous nerve terminal degeneration in the human skin. Acta Neuropathol (2001) 102(5):455–61. 10.1007/s004010100397 11699558

[118] Shun CT Chang YC Wu HP Hsieh SC Lin WM Lin YH. Skin denervation in type 2 diabetes: correlations with diabetic duration and functional impairments. Brain (2004) 127(Pt 7):1593–605. 10.1093/brain/awh180 15128619

[119] Lauria G Morbin M Lombardi R Borgna M Mazzoleni G Sghirlanzoni A. Axonal swellings predict the degeneration of epidermal nerve fibers in painful neuropathies. Neurology (2003) 61(5):631. 10.1212/01.WNL.0000070781.92512.A4 12963753

[120] Sorensen L Molyneaux L Yue DK. The relationship among pain, sensory loss, and small nerve fibers in diabetes. Diabetes Care (2006) 29(4):883–7. 10.2337/diacare.29.04.06.dc05-2180 16567832

[121] Løseth S Stålberg EV Lindal S Olsen E Jorde R Mellgren SI. Small and large fiber neuropathy in those with type 1 and type 2 diabetes: a 5-year follow-up study. J Peripheral Nervous System (2016) 21(1):15–21. 10.1111/jns.12154 26663481

[122] Malik RA Veves A Tesfaye S Smith G Cameron N Zochodne D. Small fibre neuropathy: role in the diagnosis of diabetic sensorimotor polyneuropathy. Diabetes Metab Res Rev (2011) 27(7):678–84. 10.1002/dmrr.1222 21695760

[123] Illigens BMW Gibbons CH. Sweat testing to evaluate autonomic function. Clin Autonomic Res (2008) 19(2):79–87. 10.1007/s10286-008-0506-8 PMC3046462 18989618

[124] Tsapas A Liakos A Paschos P Karagiannis T Bekiari E Tentolouris N. A simple plaster for screening for diabetic neuropathy: a diagnostic test accuracy systematic review and meta-analysis. Metabolism (2014) 63(4):584–92. 10.1016/j.metabol.2013.11.019 24405753

[125] Tavakoli M Quattrini C Begum P Fadavi H Boulton A Malik R. Neuropad and corneal confocal microscopy: new indicators for human diabetic neuropathy. Diabetologia (2010) 53(S1112):443–4.

[126] Ziegler D. Untersuchung von Polymorphismen in Kandidatengenen für periphere und kardiale autonome Neuropathie in einer bevölkerungsbezogenen Kohorte–Hans-Christian-Hagedorn-Projektförderung 2020–eine Kurzübersicht des Geförderten Dan Ziegler. Diabetologie und Stoffwechsel (2020) 15(06):450–3. 10.1055/a-1247-1356

[127] Ishibashi F Kojima R Kawasaki A Yamanaka E Kosaka A Uetake H. Correlation between sudomotor function, sweat gland duct size and corneal nerve fiber pathology in patients with type 2 diabetes mellitus. J Diabetes Invest (2014) 5(5):588–96. 10.1111/jdi.12171 PMC4188118 25411628

[128] Papanas N Giassakis G Papatheodorou K Papazoglou D Monastiriotis C Christakidis D. Use of the New Indicator Test (Neuropad ^®^) for the Assessment of the Staged Severity of Neuropathy in Type 2 Diabetic Patients. Exp Clin Endocrinol diabetes: Off J German Soc Endocrinol [and] German Diabetes Assoc (2007) 115:58–61. 10.1055/s-2007-955098 17286238

[129] Manes C Papanas N Exiara T Katsiki N Papantoniou S Kirlaki E. The indicator test Neuropad in the assessment of small and overall nerve fibre dysfunction in patients with type 2 diabetes: a large multicentre study. Exp Clin Endocrinol Diabetes (2014) 122(3):195–9. 10.1055/s-0034-1367061 24643697

[130] Papanas N Boulton AJ Malik RA Manes C Schnell O Spallone V. A simple new non-invasive sweat indicator test for the diagnosis of diabetic neuropathy. Diabetes Med (2013) 30(5):525–34. 10.1111/dme.12000 22924579

[131] Spallone V Morganti R Siampli M Fedele T D’Amato C Cacciotti L. Neuropad as a diagnostic tool for diabetic autonomic and sensorimotor neuropathy. Diabetic Med (2009) 26(7):686–92. 10.1111/j.1464-5491.2009.02760.x 19573117

[132] Mayaudon H Miloche PO Bauduceau B. A new simple method for assessing sudomotor function: Relevance in type 2 diabetes. Diabetes Metab (2010) 36(6, Part 1):450–4. 10.1016/j.diabet.2010.05.004 20739207

[133] Gin H Baudoin R Raffaitin CH Rigalleau V Gonzalez C. Non-invasive and quantitative assessment of sudomotor function for peripheral diabetic neuropathy evaluation. Diabetes Metab (2011) 37(6):527–32. 10.1016/j.diabet.2011.05.003 21715211

[134] Bordier L Dolz M Monteiro L Névoret M-L Calvet J-H Bauduceau B. Accuracy of a Rapid and Non-Invasive Method for the Assessment of Small Fiber Neuropathy Based on Measurement of Electrochemical Skin Conductances. Front Endocrinol (2016) 7:7–18. 10.3389/fendo.2016.00018 PMC4770015 26973597

[135] Mao F Liu S Qiao X Zheng H Xiong Q Wen J. Sudoscan is an effective screening method for asymptomatic diabetic neuropathy in Chinese type 2 diabetes mellitus patients. J Diabetes Investig (2017) 8(3):363–8. 10.1111/jdi.12575 PMC5415453 27607763

[136] Vinik AI Smith AG Singleton JR Callaghan B Freedman BI Tuomilehto J. Normative Values for Electrochemical Skin Conductances and Impact of Ethnicity on Quantitative Assessment of Sudomotor Function. Diabetes Technol Ther (2016) 18(6):391–8. 10.1089/dia.2015.0396 27057778

[137] Novak P. Electrochemical Skin Conductance Correlates with Skin Nerve Fiber Density. Front Aging Neurosci (2016) 8:199. 10.3389/fnagi.2016.00199 27605912 PMC4995214

[138] Rajan S Campagnolo M Callaghan B Gibbons CH. Sudomotor function testing by electrochemical skin conductance: does it really measure sudomotor function? Clin Autonomic Res (2019) 29(1):31–9. 10.1007/s10286-018-0540-0 29956008

[139] Ponirakis G Odriozola MN Odriozola S Petropoulos IN Azmi S Fadavi H. NerveCheck: An inexpensive quantitative sensory testing device for patients with diabetic neuropathy. Diabetes Res Clin Pract (2016) 113:101–7. 10.1016/j.diabres.2015.12.023 PMC5303576 26830855

[140] Yarnitsky D Granot M Nahman-Averbuch H Khamaisi M Granovsky Y. Conditioned pain modulation predicts duloxetine efficacy in painful diabetic neuropathy. PAIN (2012) 153(6):1193–8. 10.1016/j.pain.2012.02.021 22480803

[141] Rolke R Magerl W Campbell KA Schalber C Caspari S Birklein F. Quantitative sensory testing: a comprehensive protocol for clinical trials. Eur J Pain (2006) 10(1):77–88. 10.1016/j.ejpain.2005.02.003 16291301

[142] Rolke R Baron R Maier C Tölle TR Treede RD Beyer A. Quantitative sensory testing in the German Research Network on Neuropathic Pain (DFNS): Standardized protocol and reference values. Pain (2006) 123(3):231–43. 10.1016/j.pain.2006.01.041 16697110

[143] Magerl W Krumova EK Baron R Tölle T Treede R-D Maier C. Reference data for quantitative sensory testing (QST): Refined stratification for age and a novel method for statistical comparison of group data. Pain (2010) 151(3):598–605. 10.1016/j.pain.2010.07.026 20965658

[144] Dyck PJ Dyck PJB Kennedy WR Kesserwani H Melanson M Ochoa J. Limitations of quantitative sensory testing when patients are biased toward a bad outcome. Neurology (1998) 50(5). 10.1212/WNL.50.5.1213 9595965

[145] Dyck PJ Bushek W Spring EM Karnes JL Litchy WJ O’Brien PC. Vibratory and cooling detection thresholds compared with other tests in diagnosing and staging diabetic neuropathy. Diabetes Care (1987) 10(4):432–40. 10.2337/diacare.10.4.432 3622200

[146] Backonja MM Attal N Baron R Bouhassira D Drangholt M Dyck PJ. Value of quantitative sensory testing in neurological and pain disorders: NeuPSIG consensus. Pain (2013) 154(9):1807–19. 10.1016/j.pain.2013.05.047 23742795

[147] Attal N Fermanian C Fermanian J Lanteri-Minet M Alchaar H Bouhassira D. Neuropathic pain: are there distinct subtypes depending on the aetiology or anatomical lesion? Pain (2008) 138(2):343–53. 10.1016/j.pain.2008.01.006 18289791

[148] Attal N Bouhassira D Gautron M Vaillant JN Mitry E Lepère C. Thermal hyperalgesia as a marker of oxaliplatin neurotoxicity: A prospective quantified sensory assessment study. Pain (2009) 144(3):245–52. 10.1016/j.pain.2009.03.024 19457614

[149] Malmström EM Stjerna J Högestätt ED Westergren H. Quantitative sensory testing of temperature thresholds: Possible biomarkers for persistent pain? J Rehabil Med (2016) 48(1):43–7. 10.2340/16501977-2024 26450179

[150] Price RC Asenjo JF Christou NV Backman SB Schweinhardt P. The role of excess subcutaneous fat in pain and sensory sensitivity in obesity. Eur J Pain (2013) 17(9):1316–26. 10.1002/j.1532-2149.2013.00315.x 23576531

[151] Meier PM Berde CB DiCanzio J Zurakowski D Sethna NF. Quantitative assessment of cutaneous thermal and vibration sensation and thermal pain detection thresholds in healthy children and adolescents. Muscle Nerve (2001) 24(10):1339–45. 10.1002/mus.1153 11562914

[152] Lysy Z Lovblom LE Halpern EM Ngo M Ng E Orszag A. Measurement of cooling detection thresholds for identification of diabetic sensorimotor polyneuropathy in type 1 diabetes. PloS One (2014) 9(9): e106995. 10.1371/journal.pone.0106995 25216179 PMC4162569

[153] Krämer HH Rolke R Bickel A Birklein F. Thermal thresholds predict painfulness of diabetic neuropathies. Diabetes Care (2004) 27(10):2386–91. 10.2337/diacare.27.10.2386 15451905

[154] Müller LJ Marfurt CF Kruse F Tervo TMT. Corneal nerves: structure, contents and function. Exp Eye Res (2003) 76(5):521–42. 10.1016/S0014-4835(03)00050-2 12697417

[155] Marfurt C Anokwute MC Fetcko K Mahony-Perez E Farooq H Ross E. Comparative Anatomy of the Mammalian Corneal Subbasal Nerve Plexus. Invest Ophthalmol Vis Sci (2019) 60(15):4972–84. 10.1167/iovs.19-28519 PMC6886725 31790560

[156] Kalteniece A Ferdousi M Petropoulos I Azmi S Adam S Fadavi H. Greater corneal nerve loss at the inferior whorl is related to the presence of diabetic neuropathy and painful diabetic neuropathy. Sci Rep (2018) 8(1):3283. 10.1038/s41598-018-21643-z 29459766 PMC5818543

[157] Tavakoli M Petropoulos IN Malik RA. Corneal confocal microscopy to assess diabetic neuropathy: an eye on the foot. J Diabetes Sci Technol (2013) 7(5):1179–89. 10.1177/193229681300700509 PMC3876361 24124944

[158] Tavakoli M Hossain P Malik RA. Clinical applications of corneal confocal microscopy. Clin Ophthalmol (Auckland NZ) (2008) 2(2):435–45. 10.2147/OPTH.S1490 PMC2693976 19668734

[159] Hertz P Bril V Orszag A Ahmed A Ng E Nwe P. Reproducibility of *in vivo* corneal confocal microscopy as a novel screening test for early diabetic sensorimotor polyneuropathy. Diabetes Med (2011) 28(10):1253–60. 10.1111/j.1464-5491.2011.03299.x 21434993

[160] Oliveira-Soto L Efron N. Morphology of corneal nerves using confocal microscopy. Cornea (2001) 20(4):374–84. 10.1097/00003226-200105000-00008 11333324

[161] Maddaloni E Sabatino F Del Toro R Crugliano S Grande S Lauria Pantano A. In vivo corneal confocal microscopy as a novel non-invasive tool to investigate cardiac autonomic neuropathy in Type 1 diabetes. Diabetes Med (2015) 32(2):262–6. 10.1111/dme.12583 25251450

[162] Ishibashi F Kojima R Taniguchi M Kosaka A Uetake H Tavakoli M. The Expanded Bead Size of Corneal C-Nerve Fibers Visualized by Corneal Confocal Microscopy Is Associated with Slow Conduction Velocity of the Peripheral Nerves in Patients with Type 2 Diabetes Mellitus. J Diabetes Res (2016) 7. 10.1155/2016/3653459 PMC4987467 27563679

[163] Labbé A Alalwani H Van Went C Brasnu E Georgescu D Baudouin C. The relationship between subbasal nerve morphology and corneal sensation in ocular surface disease. Invest Ophthalmol Vis Sci (2012) 53(8):4926–31. 10.1167/iovs.11-8708 22695962

[164] del Castillo JMB Wasfy MAS Fernandez C Garcia-Sanchez J. An In Vivo Confocal Masked Study on Corneal Epithelium and Subbasal Nerves in Patients with Dry Eye. Invest Ophthalmol Visual Sci (2004) 45(9):3030–5. 10.1167/iovs.04-0251 15326117

[165] Petropoulos IN Ferdousi M Marshall A Alam U Ponirakis G Azmi S. The Inferior Whorl For Detecting Diabetic Peripheral Neuropathy Using Corneal Confocal Microscopy. Invest Ophthalmol Visual Sci (2015) 56(4):2498–504. 10.1167/iovs.14-15919 PMC4408884 25783609

[166] Kowtharapu BS Winter K Marfurt C Allgeier S Kohler B Hovakimyan M. Comparative quantitative assessment of the human corneal sub-basal nerve plexus by *in vivo* confocal microscopy and histological staining. Eye (Lond) (2017) 31(3):481–90. 10.1038/eye.2016.220 PMC5350356 27813513

[167] Brines M Culver DA Ferdousi M Tannemaat MR van Velzen M Dahan A. Corneal nerve fiber size adds utility to the diagnosis and assessment of therapeutic response in patients with small fiber neuropathy. Sci Rep (2018) 8(1). 10.1038/s41598-018-23107-w PMC5856845 29549285

[168] Chen X Graham J Dabbah MA Petropoulos IN Tavakoli M Malik RA. An Automatic Tool for Quantification of Nerve Fibers in Corneal Confocal Microscopy Images. IEEE Trans BioMed Eng (2017) 64(4):786–94. 10.1109/TBME.2016.2573642 PMC5512547 27295646

[169] Dana MR. Corneal Antigen-Presenting Cells: Diversity, Plasticity, and Disguise The Cogan Lecture. Invest Ophthalmol Visual Sci (2004) 45(3):722–7. 10.1167/iovs.03-0803 14985280

[170] Kalogeropoulos D Papoudou-Bai A Lane M Goussia A Charchanti A Moschos M. Antigen-presenting cells in ocular surface diseases. Int Ophthalmol (2020) 40:1603–18. 10.1007/s10792-020-01329-0 32107692

[171] Alzahrani Y Colorado LH Pritchard N Efron N. Longitudinal changes in Langerhans cell density of the cornea and conjunctiva in contact lens-induced dry eye. Clin Exp Optom (2017) 100(1):33–40. 10.1111/cxo.12399 27353750

[172] Resch MD Marsovszky L Németh J Bocskai M Kovács L Balog A. Dry eye and corneal langerhans cells in systemic lupus erythematosus. J Ophthalmol (2015) 2015:543835. 10.1155/2015/543835 25893112 PMC4393942

[173] Machetta F Fea AM Actis AG de Sanctis U Dalmasso P Grignolo FM. In vivo confocal microscopic evaluation of corneal langerhans cells in dry eye patients. Open Ophthalmol J (2014) 8:51–9. 10.2174/1874364101408010051 PMC4195179 25317216

[174] Su P-Y Hu F-R Chen Y-M Han J-H Chen W-L. Dendritiform Cells Found in Central Cornea by In-Vivo Confocal Microscopy in a Patient with Mixed Bacterial Keratitis. Ocular Immunol inflammation (2006) 14:241–4. 10.1080/09273940600732398 16911987

[175] Wu LQ Cheng JW Cai JP Le QH Ma XY Gao LD. Observation of Corneal Langerhans Cells by In Vivo Confocal Microscopy in Thyroid-Associated Ophthalmopathy. Curr Eye Res (2016) 41(7):927–32. 10.3109/02713683.2015.1133833 26735162

[176] Zhivov A Stave J Vollmar B Guthoff R. In vivo confocal microscopic evaluation of Langerhans cell density and distribution in the normal human corneal epithelium. Graefe’s Arch Clin Exp Ophthalmology (2005) 243(10):1056–61. 10.1007/s00417-004-1075-8 15856272

[177] Tavakoli M Boulton AJM Efron N Malik RA. Increased Langerhan cell density and corneal nerve damage in diabetic patients: role of immune mechanisms in human diabetic neuropathy. Contact lens anterior eye: J Br Contact Lens Assoc (2011) 34(1):7–11. 10.1016/j.clae.2010.08.007 PMC3017662 20851037

[178] Rosenberg ME Tervo TM Immonen IJ Müller LJ Grönhagen-Riska C Vesaluoma MH. Corneal structure and sensitivity in type 1 diabetes mellitus. Invest Ophthalmol Vis Sci (2000) 41(10):2915–21. 10967045

[179] Malik RA Kallinikos P Abbott CA van Schie CHM Morgan P Efron N. Corneal confocal microscopy: a non-invasive surrogate of nerve fibre damage and repair in diabetic patients. Diabetologia (2003) 46(5):683–8. 10.1007/s00125-003-1086-8 12739016

[180] Midena E Brugin E Ghirlando A Sommavilla M Avogaro A. Corneal diabetic neuropathy: a confocal microscopy study. J Refract Surg (2006) 22:1047–52. 10.3928/1081-597X-20061102-08 17444092

[181] Bitirgen G Turkmen K Malik RA Ozkagnici A Zengin N. Corneal confocal microscopy detects corneal nerve damage and increased dendritic cells in Fabry disease. Sci Rep (2018) 8(1). 10.1038/s41598-018-30688-z PMC6095897 30116053

[182] Che N-N Yang H-Q. Potential use of corneal confocal microscopy in the diagnosis of Parkinson’s disease associated neuropathy. Transl Neurodegener (2020) 9(1). 10.1186/s40035-020-00204-3 PMC7330988 32611440

[183] Cruzat A Qazi Y Hamrah P. In Vivo Confocal Microscopy of Corneal Nerves in Health and Disease. Ocul Surf (2017) 15(1):15–47. 10.1016/j.jtos.2016.09.004 27771327 PMC5512932

[184] Tavakoli M Marshall A Banka S Petropoulos IN Fadavi H Kingston H. Corneal confocal microscopy detects small-fiber neuropathy in Charcot-Marie-Tooth disease type 1A patients. Muscle Nerve (2012) 46(5):698–704. 10.1002/mus.23377 22996176 PMC3469745

[185] Tavakoli M Marshall A Thompson L Kenny M Waldek S Efron N. Corneal confocal microscopy: A novel noninvasive means to diagnose neuropathy in patients with fabry disease. Muscle Nerve (2009) 40(6):976–84. 10.1002/mus.21383 19902546

[186] Tavakoli M Mitu-Pretorian M Petropoulos IN Fadavi H Asghar O Alam U. Corneal confocal microscopy detects early nerve regeneration in diabetic neuropathy after simultaneous pancreas and kidney transplantation. Diabetes (2013) 62(1):254–60. 10.2337/db12-0574 PMC3526062 23002037

[187] Halpern EM Lovblom LE Orlov S Ahmed A Bril V Perkins BA. The impact of common variation in the definition of diabetic sensorimotor polyneuropathy on the validity of corneal *in vivo* confocal microscopy in patients with type 1 diabetes: a brief report. J Diabetes its Complications (2013) 27(3):240–2. 10.1016/j.jdiacomp.2012.10.011 23266297

[188] Tavakoli M Ferdousi M Petropoulos IN Morris J Pritchard N Zhivov A. Normative values for corneal nerve morphology assessed using corneal confocal microscopy: a multinational normative data set. Diabetes Care (2015) 38(5):838–43. 10.2337/dc14-2311 PMC4407754 25633665

[189] Pritchard N Edwards K Dehghani C Fadavi H Jeziorska M Marshall A. Longitudinal assessment of neuropathy in type 1 diabetes using novel ophthalmic markers (LANDMark): study design and baseline characteristics. Diabetes Res Clin Pract (2014) 104(2):248–56. 10.1016/j.diabres.2014.02.011 24629408

[190] Edwards K Pritchard N Vagenas D Russell A Malik RA Efron N. Utility of corneal confocal microscopy for assessing mild diabetic neuropathy: baseline findings of the LANDMark study. Clin Exp Optometry (2012) 95(3):348–54. 10.1111/j.1444-0938.2012.00740.x 22540156

[191] Ziegler D Papanas N Zhivov A Allgeier S Winter K Ziegler I. Early detection of nerve fiber loss by corneal confocal microscopy and skin biopsy in recently diagnosed type 2 diabetes. Diabetes (2014) 63(7):2454–63. 10.2337/db13-1819 24574045

[192] Asghar O Petropoulos IN Alam U Jones W Jeziorska M Marshall A. Corneal confocal microscopy detects neuropathy in subjects with impaired glucose tolerance. Diabetes Care (2014) 37(9):2643–6. 10.2337/dc14-0279 PMC4140158 24969581

[193] Kawamoto K Chikama T Takahashi N Nishida T. In vivo observation of Langerhans cells by laser confocal microscopy in Thygeson’s superficial punctate keratitis. Mol Vis (2009) 15:1456–62. PMC2718741 19649162

[194] Dauch JR Bender DE Luna-Wong LA Hsieh W Yanik BM Kelly ZA. Neurogenic factor-induced Langerhans cell activation in diabetic mice with mechanical allodynia. J Neuroinflamm (2013) 10:64. 10.1186/1742-2094-10-64 PMC3685572 23672639

[195] Davidson EP Coppey LJ Holmes A Lupachyk S Dake BL Oltman CL. Characterization of diabetic neuropathy in the Zucker diabetic Sprague-Dawley rat: a new animal model for type 2 diabetes. J Diabetes Res (2014) e714273. 10.1155/2014/714273 PMC4211210 25371906

[196] Leppin K Behrendt AK Reichard M Stachs O Guthoff RF Baltrusch S. Diabetes mellitus leads to accumulation of dendritic cells and nerve fiber damage of the subbasal nerve plexus in the cornea. Invest Ophthalmol Vis Sci (2014) 55(6):3603–15. 10.1167/iovs.14-14307 24781935

[197] Hamrah P Zhang Q Liu Y Dana MR. Novel characterization of MHC class II-negative population of resident corneal Langerhans cell-type dendritic cells. Invest Ophthalmol Vis Sci (2002) 43(3):639–46. 11867578

[198] Ferdousi M Romanchuk K Mah JK Virtanen H Millar C Malik RA. Early corneal nerve fibre damage and increased Langerhans cell density in children with type 1 diabetes mellitus. Sci Rep (2019) 9(1):8758. 10.1038/s41598-019-45116-z 31217448 PMC6584636

[199] Basantsova NY Starshinova AA Dori A Zinchenko YS Yablonskiy PK Shoenfeld Y. Small-fiber neuropathy definition, diagnosis, and treatment. Neurol Sci (2019) 40(7):1343–50. 10.1007/s10072-019-03871-x 30968230

[200] Halpern EM Lovblom LE Orlov S Bril V Perkins BA eds. The existence of rapid small fiber decline in type 1 diabetes: a potential proxy for neuropathy progression using in vivo corneal confocal microscopy. In: 22nd Annual Meeting of the Diabetic Neuropathy Study Group. Dresden, Germany. (2013)

[201] Edwards K Pritchard N Dehghani C Vagenas D Russell A Malik RA. Corneal confocal microscopy best identifies the development and progression of neuropathy in patients with type 1 diabetes. J Diabetes Complications (2017) 31(8):1325–7. 10.1016/j.jdiacomp.2017.04.025 28551295

[202] Azmi S Ferdousi M Petropoulos IN Ponirakis G Alam U Fadavi H. Corneal Confocal Microscopy Identifies Small-Fiber Neuropathy in Subjects With Impaired Glucose Tolerance Who Develop Type 2 Diabetes. Diabetes Care (2015) 38(8):1502–8. 10.2337/dc14-2733 PMC4512140 25877814

[203] Tavakoli M Kallinikos P Iqbal A Herbert A Fadavi H Efron N. Corneal confocal microscopy detects improvement in corneal nerve morphology with an improvement in risk factors for diabetic neuropathy. Diabetic Med: J Br Diabetic Assoc (2011) 28(10):1261–7. 10.1111/j.1464-5491.2011.03372.x PMC3181044 21699561

[204] Mehra S Tavakoli M Kallinikos PA Efron N Boulton AJ Augustine T. Corneal confocal microscopy detects early nerve regeneration after pancreas transplantation in patients with type 1 diabetes. Diabetes Care (2007) 30(10):2608–12. 10.2337/dc07-0870 17623821

[205] Lewis EJH Perkins BA Lovblom LE Bazinet RP Wolever TMS Bril V. Effect of omega-3 supplemensstation on neuropathy in type 1 diabetes: A 12-month pilot trial. Neurology (2017) 88(24):2294–301. 10.1212/WNL.0000000000004033 PMC5567321 28515269

[206] Brines M Dunne AN van Velzen M Proto PL Ostenson C-G Kirk RI. ARA 290, a nonerythropoietic peptide engineered from erythropoietin, improves metabolic control and neuropathic symptoms in patients with type 2 diabetes. Mol Med (Cambridge Mass) (2015) 20(1):658–66. 10.2119/molmed.2014.00215 PMC4365069 25387363

